# Transfer of Complex Skill Learning from Virtual to Real Rowing

**DOI:** 10.1371/journal.pone.0082145

**Published:** 2013-12-20

**Authors:** Georg Rauter, Roland Sigrist, Claudio Koch, Francesco Crivelli, Mark van Raai, Robert Riener, Peter Wolf

**Affiliations:** 1 Sensory-Motor Systems (SMS) Lab, Institute of Robotics and Intelligent Systems (IRIS), ETH Zurich, Zurich, Switzerland; 2 Medical Faculty, University of Zurich, Zurich, Switzerland; Hungarian Academy of Sciences, Hungary

## Abstract

Simulators are commonly used to train complex tasks. In particular, simulators are applied to train dangerous tasks, to save costs, and to investigate the impact of different factors on task performance. However, in most cases, the transfer of simulator training to the real task has not been investigated. Without a proof for successful skill transfer, simulators might not be helpful at all or even counter-productive for learning the real task. In this paper, the skill transfer of complex technical aspects trained on a scull rowing simulator to sculling on water was investigated. We assume if a simulator provides high fidelity rendering of the interactions with the environment even without augmented feedback, training on such a realistic simulator would allow similar skill gains as training in the real environment. These learned skills were expected to transfer to the real environment. Two groups of four recreational rowers participated. One group trained on water, the other group trained on a simulator. Within two weeks, both groups performed four training sessions with the same licensed rowing trainer. The development in performance was assessed by quantitative biomechanical performance measures and by a qualitative video evaluation of an independent, blinded trainer. In general, both groups could improve their performance on water. The used biomechanical measures seem to allow only a limited insight into the rowers' development, while the independent trainer could also rate the rowers' overall impression. The simulator quality and naturalism was confirmed by the participants in a questionnaire. In conclusion, realistic simulator training fostered skill gains to a similar extent as training in the real environment and enabled skill transfer to the real environment. In combination with augmented feedback, simulator training can be further exploited to foster motor learning even to a higher extent, which is subject to future work.

## Introduction

Training simulators are commonly used to prevent accidents and injuries in potentially dangerous tasks, to increase training time, to enhance training quality by the application of sensor technology and provision of augmented feedback, or to adjust conditions to the user's skill level.— The term “skill” is used as an abbreviation for “motor skill” throughout this paper: “A skill for which the primary determinant of success is the quality of the movement that the performer produces” [1].— In addition, simulators help to save costs, or to investigate the impact of different intrinsic and extrinsic factors on the task performance, e.g. in training of supervising nuclear power plants [2], military operations [3], [4], navigation [5]–[7], or surgery [8]–[11]. Due to the constantly increasing performance of today's computers, simulations of more and more complex motor tasks are possible, i.e. tasks that “cannot be mastered in a single session, have several degrees of freedom, and perhaps tend to be ecologically valid” [12]. This possibility enables the examination of the questionable generalization of conclusions drawn on simple laboratory tasks— which have been investigated mainly so far— to complex tasks [13]. During training, the signals that are measured to drive the simulation can be used to evaluate the trainee's performance. Consequently, simulators can feature a comprehensive documentation of the learning progress of a trainee [14], [15].— By learning, we consequently refer to “motor learning”: “Changes in internal processes that determine a person's capability for producing a motor task. The level of a person's motor learning improves with practice and is often inferred by observing relatively stable levels of the person's motor performance” [1].— Moreover, simulators enable studies determining the most effective augmented feedback during task execution. For example, augmented audiovisual feedback was found to be effective in flight tasks [16], [17] and in a driving task [18]. Simulators that involve augmented reality or virtual reality are assumed to hold great potential to foster motor learning [19]. Accordingly, quite a few sport simulators have been developed, e.g. in golf [20], tennis [21], football [22], rowing [23]–[25], canoeing [26], bicycling [27], [28], bobsledding [29], archery [30], gymnastics [31], and dancing [32], [33]. However, their effectiveness on motor learning has hardly been investigated. The effectiveness of a training simulator is given, when skills learnt on the simulator can be transferred to the real environment [34]. So far, investigations of transferability might have been hampered due to limited resources, technical constraints, or safety restrictions, or because of too heavy and too bulky measurement systems required assessing the performance under real conditions. Also, the acceptance to apply measurement systems in real environments may be low, when their application exceeds a certain degree of complexity or takes too long.

In sports, the likely first transfer study focused on simulator training for playing table tennis. Participants trained specific table tennis shots either with an expert coach (control group) or in a simulator (experimental group). The simulator applied audiovisual rendering and audiovisual augmented feedback. In retention tests on the real task, the simulator group significantly outperformed the control group [35]. However, as soon as the visual cue of the approaching ball was not present in the virtual environment, the simulator group could not transfer the gained skills from training to the real task. Key features are crucial during structural learning, i.e. the development of a movement plan. During structural learning the athletes learn the relationships between input, e.g. motor commands, and output, e.g. racket motion, for the desired behavior [36]. Consequently, a modification of real motor sensory interactions (or key features) will limit the transferability of training simulators and, thus, their impact on skill learning. In another transfer study on juggling, novice participants trained either in the real environment (control group) or in the real environment alternating with juggling in a simulator (experimental group). The simulator allowed juggling at usual speeds as well as at speeds lower than in reality. After ten days of training, both groups reached a similar level of performance in terms of consecutive juggling cycles. At a higher juggling speed, the experimental group outperformed the control group although simulator training was only based on rendering with modified physical parameters, i.e. motor and sensor fidelity of the simulator was low. Therefore, the authors concluded that cognitive aspects of the task might have been better learnt due to additional simulator training [37]. Interestingly, the juggling performance seems to depend more on training of cognitive aspects enhancing hand-eye coordination than on training of realistic sensations through haptic interactions with the environment. Thus, as long as the key features of a task are well represented in the simulation, modifications of physical parameters or simplifications in the visualization of the task can supplement conventional training.

In sports like table tennis and juggling, the focus lies on “hand-eye” coordination of movements, whereby haptic interaction forces are mainly experienced at discrete time points and transfer comparatively little energy from the athlete to the ball or vice versa. This is different for sports like rowing: Haptic interaction forces between rower, boat, oar, and water are continuously present, and a comparatively large amount of energy is needed to reach high boat velocities. More precisely, a high mean boat velocity can only be reached, if the rower is able to apply high forces in travelling direction over a maximized oar movement in water (maximized stroke length) at a high stroke rate. The combination of a well-coordinated dynamic movement while efficiently producing maximal forward propulsion of the boat characterizes a good rowing technique. For this reason, the demands on a realistic rowing simulation are not only the rendering of visual and auditory interactions with the environment, but also the realistic rendering of haptic interactions between oar and water.

The currently most economical solution to render oar-water interactions is realized in indoor rowing ergometers such as the *RowPerfect* ® (Rowperfect Pty Ltd, Harbord, NSW, Australia), or the *Concept2 ®* ergometers (Concept2 Deutschland GmbH, Hamburg, Germany). Here, resistive oar-water forces are rendered by a cable-driven windmill. The felt resistance does not depend on the height at which the cable is pulled out of the windmill, but on the cable's velocity. Thus, the rower cannot develop a feeling for the height at which the oars are immersed into water. Furthermore, the rower cannot train oar handling aspects since the cable is bimanually pulled out of the windmill with a rod [38]. Training of proper oar handling is possible on rowing devices such as the *OarTec®* (OarTec, Sydney, NSW, Australia), or the *Swingulator* ® (Rowing Innovations, Williston, Vermont, USA). A more sophisticated scull simulator was presented by researchers from Pisa. Athletes could train in a virtual reality scenario with a passive haptic device allowing independent handling of two oars. The oar handles were connected to flywheel dissipators, which generated oar angle- and oar velocity-dependent water resistances when the oar was pulled through the virtual water in the correct direction [23]. The highest level of realism concerning oar-water interaction forces is provided in tank rowing facilities. In rowing tanks, rowers can perform rowing training indoors while a rowing trainer can stand next to them to provide feedback. Tank rowing was shown to deliver more complete and rowing specific power data than a rowing ergometer. Furthermore, tank rowing can provide objective, rowing specific data to analyze technical aspects such as oar handling, technical efficiency, consistency, stroke rate, etc. [39]. However, such facilities are stationary, power intensive due to large pumps, extremely costly and have to be maintained constantly.

**Figure 1:**
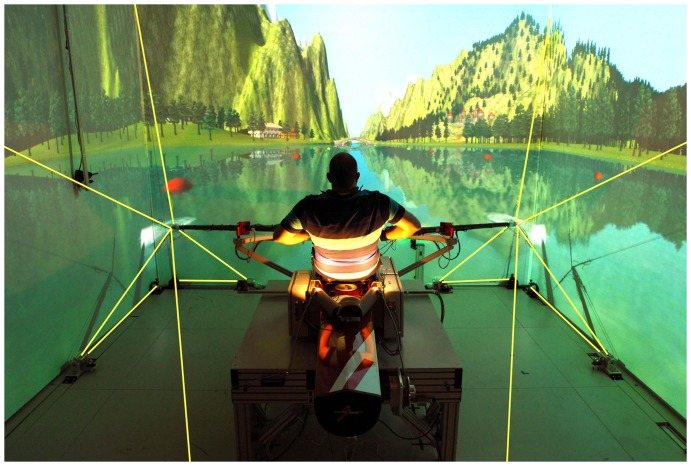
Training in the scull rowing simulator of the SMS-Lab at ETH Zurich. This scull rowing simulator is set up in a CAVE comprising auditory-, visual-, and haptic displays.

In our lab at ETH Zurich, we have set up a CAVE (Cave Automated Virtual Environment) for training and investigations of manifold applications using multimodal rendering and also augmented feedback, i.e. extrinsic feedback that provides information beyond deceptively realistic simulation of the environment. We have realized a tennis application [40], [41], a platform for sleep research [41], and a sweep rowing simulator [25], [41]–[44]. Based on our previous work, literature [45]–[47], and input from professional rowing experts, we were able to extend our sweep rowing simulator to a scull rowing simulator. Inside this simulator, rowers can feel the water level, remark the buoyancy forces of the oars in water, feel force changes at the oars due to turned oar blades and inertia effects of the rower and boat, sense a boat speed-dependent air flow, and row in any direction or turn the boat (virtually), which is even not possible in a tank rowing device (Figure 1).

According to D. Gopher (2012), the following four points should be addressed to assess the value of a multimodal virtual environment platform for skill acquisition: “1) A comparative evaluation of the differential experience of performing the same tasks on the VR platform and in the real world; 2) Evaluation of the contribution of accelerators; 3) Assessment of training protocols that will maximize learning and skill acquisition on a platform; 4) Transfer of training studies.” [34]. We already assessed the value of our simulator, e.g. in one study, the high realism of our rowing simulator was indicated for sweep rowing on a theoretical basis and by positive feedback from rowing experts [25]. The simulator's realism was further confirmed by a second study showing that professional rowers could approach their individual maximal mean boat velocity closer than recreational rowers due to a better rowing technique [44]. In addition, we have evaluated the effectiveness of different augmented feedback designs and modalities to accelerate learning of a rowing-type movement on the simulator [48]. Combining such augmented feedback with training protocols that maximize learning seem to be particularly valuable when transfer to the real task is given even when no additional learning accelerators are used in the simulator. Thus, the goal of the current study was to determine skill transfer from simulator training to on-water rowing while just the rowing scenario was rendered.

Eight intermediate rowers trained either exclusively on the simulator (simulator group) or exclusively on water (on-water group = control group). Both groups received four trainings with the same licensed rowing trainer. Physical parameters in the simulator were set to values measured in real rowing. The skill transfer/development was assessed quantitatively through biomechanical performance measures and qualitatively through video analysis by an independent and blinded rowing trainer (other than the trainer that guided the training).

Due to the high level of realism given by our scull simulator, we expected that the simulator group could transfer improved skills to rowing on water and that rowers of both groups could improve their individual rowing skills to a similar extent on water. This expectation was finally confirmed by the independent rowing trainer. Furthermore, all participants claimed their personal profit from the study.

## Methods

### Phases of a Rowing Cycle

**Figure 2:**
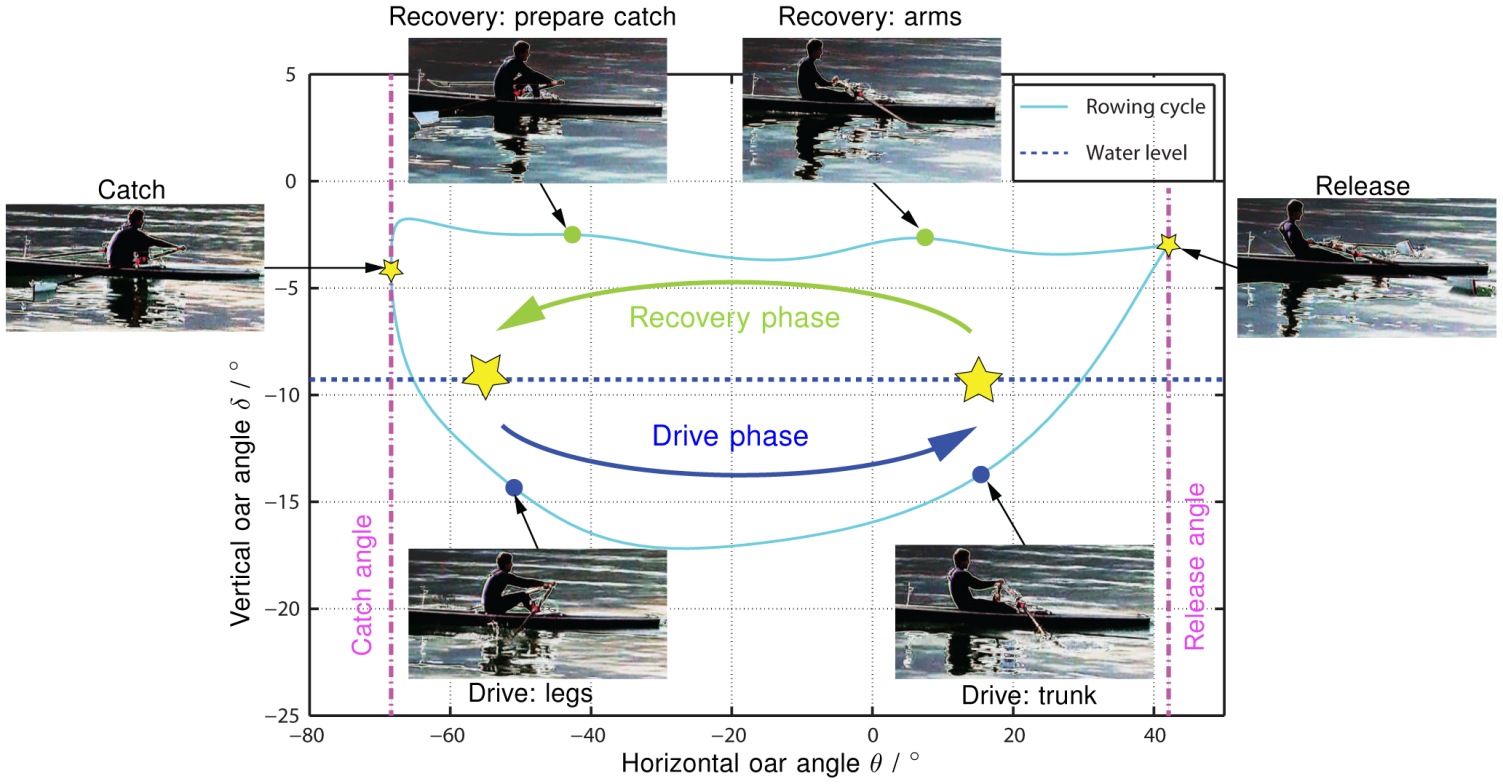
The phases of the rowing cycle. The rowing cycle is divided into two phases: the recovery phase, where the oar is in water and the drive phase, where the oar is pulled through water. The stars indicate the locations, where the phases are separated. These are the points, where the oar enters water (catch) and where the oar is lifted out of water (release).

Rowing is a periodic movement that can be divided into cycles, i.e. strokes. Each rowing cycle starts at the catch, followed by the drive phase, the release, and the recovery phase before it restarts at the catch (Figure 2). At the catch, the oar blades are moved vertically into the water, ready to exert propulsive forces. In the following drive phase, the rower performs a coordinatively demanding sequential movement of legs, trunk, and arms. The rower uses the power generated by this sequential movement to pull the oars through water and propel the boat. At the end of the drive phase, i.e. the release, both blades are vertically pushed out of water. The subsequent recovery phase is characterized by the sequential movement of arms, trunk, and legs to bring the oars back to a minimal horizontal angle, where another stroke is started with a new catch [47]. In principle, there are two different types of rowing: sweep rowing and scull. During sweep rowing, each rower only holds one oar with both hands, thus, an even number of rowers is needed to propel the boat. In scull rowing, as investigated in this study, the rower can propel the boat by manipulating two oars simultaneously, holding one oar in each hand.

### Scull Simulator

#### Setup

For the current study, a single scull simulator was placed in a CAVE at the Sensory-Motor Systems Lab, ETH Zurich. In the center of this CAVE, one rower was seated inside a trimmed rowing skiff and held one trimmed oar in each hand. A sound wave field synthesis system (112 speakers and 4 subwoofers, Iosono GmbH, Erfurt, Germany) placed in a ring around the CAVE at the level of the rower's ears was used to render a realistic soundscape. Furthermore, 3 screens (4 m×3 m, projectors: Projection Design F3+, Norway) surrounded the rower (Figure 1). When the rower moved the oars, he/she could hear and see the oar-water interaction that propelled the virtual boat through a virtual scenario. In addition to the visual and auditory impressions, the oar-water interaction was rendered haptically so that the rower could feel the oar-water interaction. Therefore, two custom-made tendon-based parallel robots [41], [43], [49] were connected to the end-effectors, i.e. outer ends of the trimmed oars (Figure 1).

**Figure 3:**
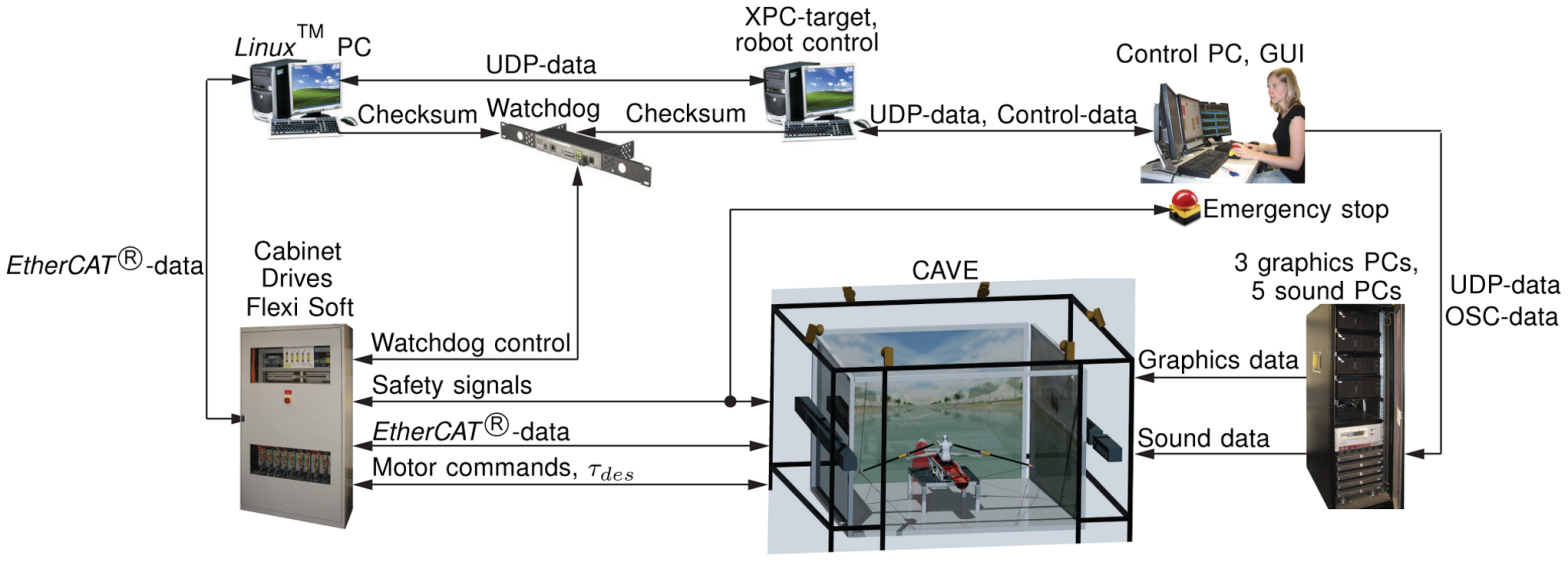
Setup and implementation of data transfer for the scull simulator. One human operator can control the entire scull simulator from the control PC. The core of the setup is the XPC-target that calculates the real-time rowing model and the haptic interaction forces. This XPC-target collects sensor data from the robot and sensors inside the CAVE, processes this data, and transfers it via different protocols to the other PCs that render sound and graphics. Safety of the simulator is constantly guaranteed by a watchdog, safety relays in the cabinet, error detection software in the XPC-target, and the human operator. Furthermore, the participant in the simulator has emergency cords around his wrists that stop the simulator as soon as the hands are too far away from the oar handles.

**Figure 4:**
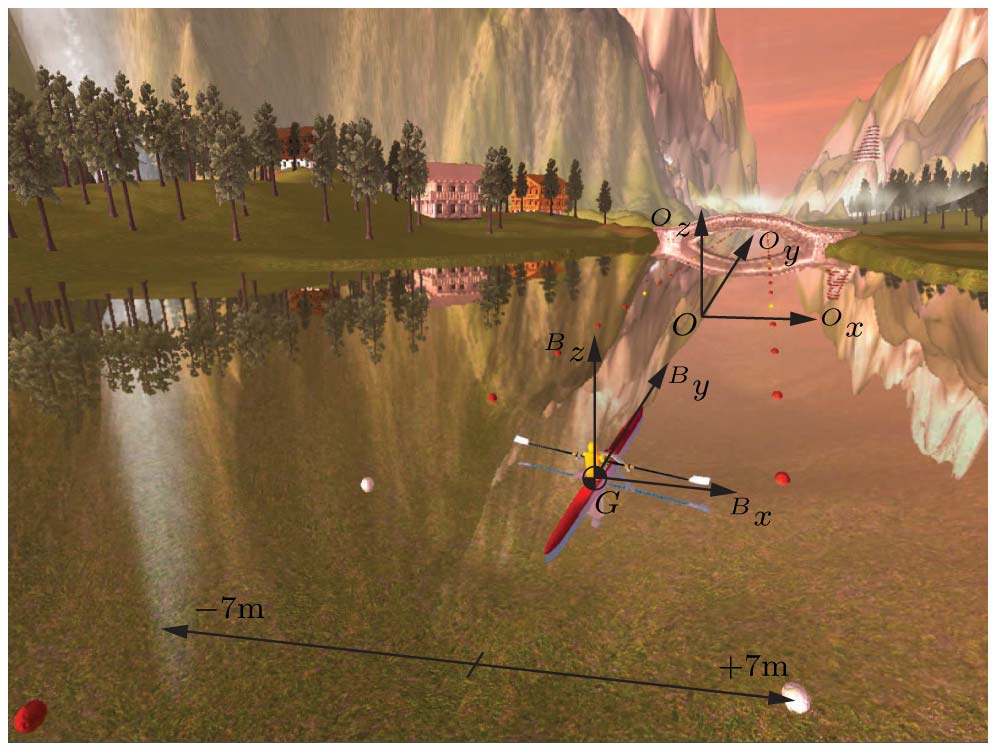
The training scenario was only used in the simulator group. This scenario provided some distraction for the participant during the four trainings, similar to a training environment on water. For rendering, a global coordinate system (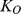) and boat coordinate system (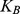) where defined. Two lines of buoys limited the rower's workspace within the scenario. In this way the rower was in a setting comparable to a rowing race and at the same time, the rower could not leave the virtual world.

**Figure 5:**
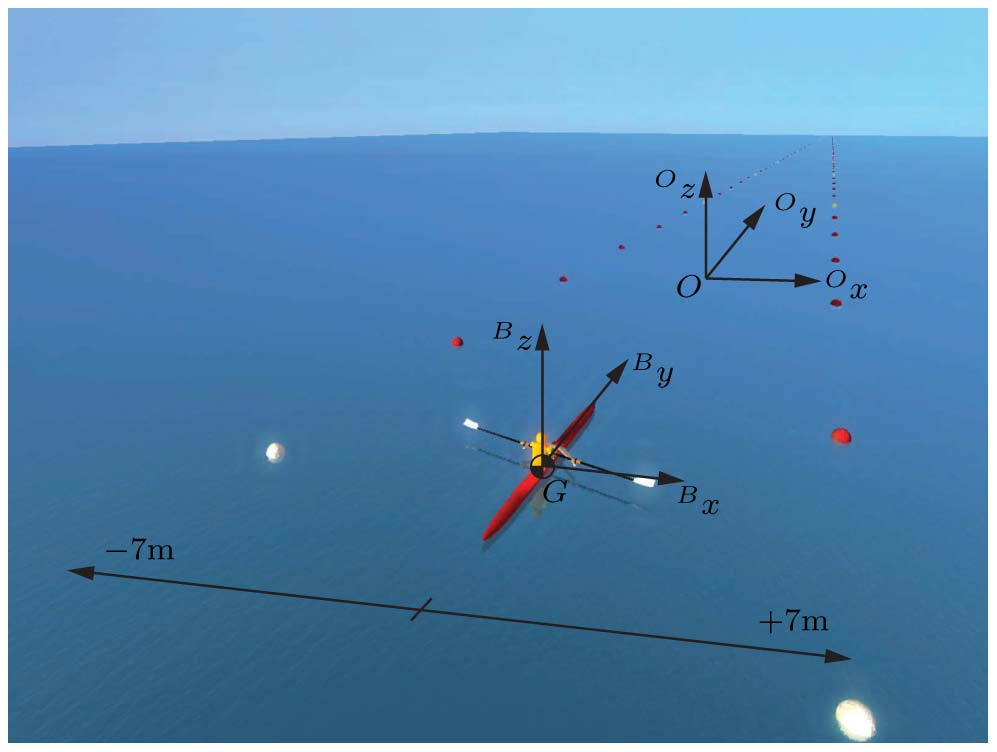
The ocean scenario was used in all participants during the baseline and retention tests. This scenario was designed to prevent the participants from distractions and to focus on their rowing technique. For rendering, a global coordinate system (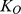) and boat coordinate system (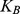) where defined. Two lines of buoys limited the rower's workspace within the scenario. In this way the rower was in a setting comparable to a rowing race and at the same time, the rower could not leave the virtual world.

In this study, both tendon-based parallel robots were composed by five drive trains, i.e. five axes per robot. Each of these drive trains consisted of a rope (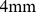 “D-Pro Dyneema”, Rosenberger Tauwerk GmbH, Lichtenberg, Germany) that linked a motorized winch located outside the CAVE over deflection units and a force sensor (K100.2k, Transmetra GmbH, Neuhausen am Rheinfall, Germany) to one oar end (Figure 1). To render the haptic interactions between oars and water, all drive trains were controlled simultaneously through one *Matlab/Simulink*® model running at 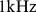 on an XPC-target. Data exchange with the sensors, drives, brakes, and safety devices of the robot was entirely handled using *EtherCAT®* -data across a *Linux*™ PC. One operator could control the entire scull simulator through a graphical user interface (GUI) written in C++ on a control PC. This GUI controlled the states of the *Matlab/Simulink®* model using control-data defined by the “XPCAPI.dll” included in the *Matlab/Simulink®* package. Furthermore, the soundscape was controlled by the GUI: UDP-data provided by the XPC-target was transformed to splash sounds of the oar-water interaction on the control PC and forwarded to the sound system via OSC-protocol. The graphical scenario was driven by the boat, rower and oar movements that were sent to each graphics PC in form of UDP-data from the XPC-target (Figure 3). The graphical scene was developed in a commercially available game engine (Unity Pro, Unity Technologies, San Francisco, USA). For the transfer study described in this paper, two different graphics scenarios were developed: a scenario with a diverse landscape to make the trainings visually attractive (Figure 4) and an ocean scenario for baseline and retention tests to avoid visual distraction (Figure 5). The update rate of both scenarios was set to 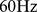 at a resolution of 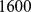
×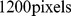. In the ocean scenario, the update rate could be kept constant, while in the more demanding training scenario, the update rate could decrease to a minimum of 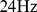 in dependence of the complexity of the rendered objects and scene depth. UDP-data for the graphics scenario was sent at 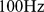. Maximal delays of the graphics data from measuring data at the robots' sensors until data arrived at the graphics PCs were 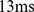.

**Figure 6:**
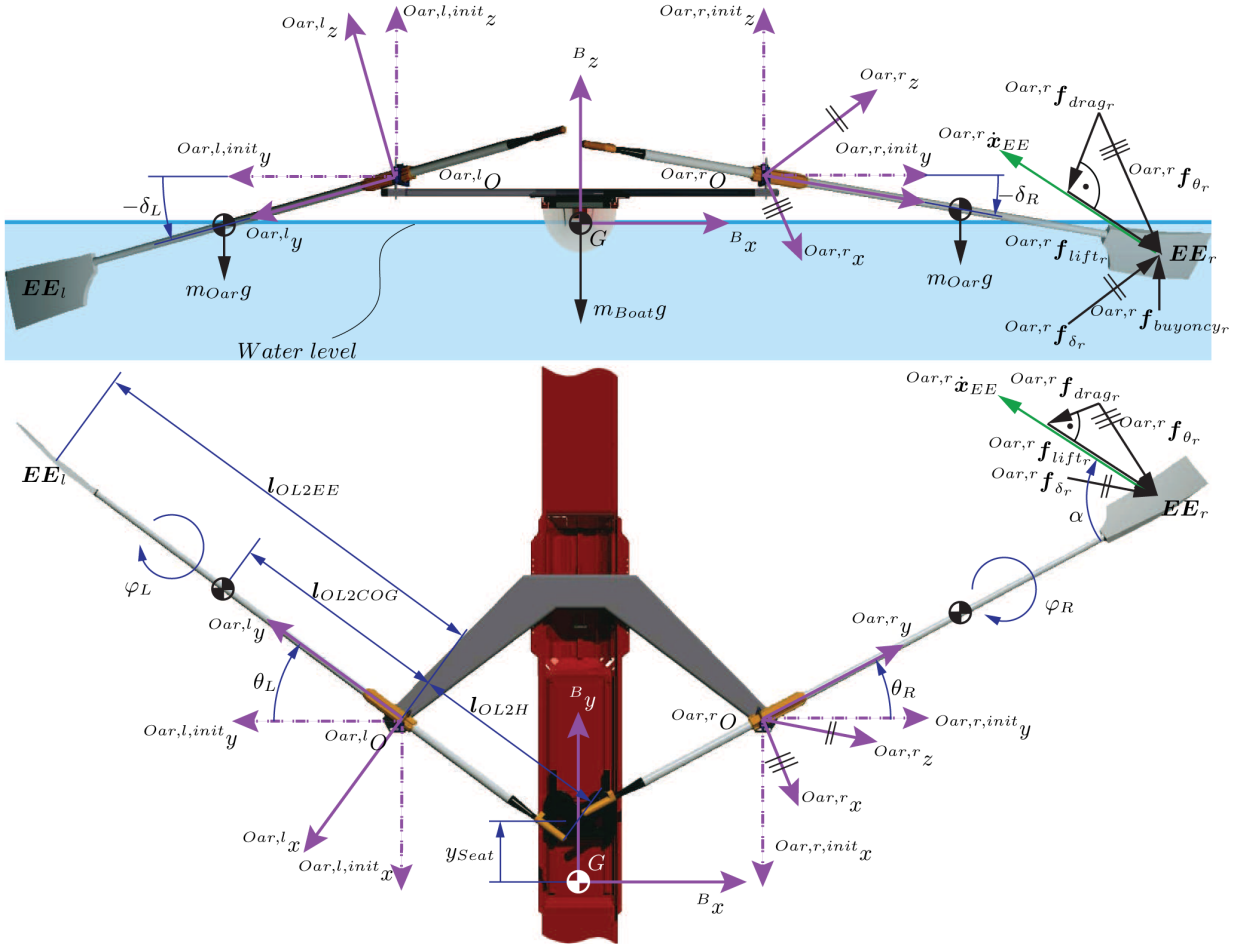
Definition of the boat's coordinate systems, the forces at the oars, the oar angles, the seat position, and centers of gravity.

To render the haptic interaction between the oars and the water, the oar movements induced by the rower have to be known. Therefore, the end-effector positions of both oars were determined by forward kinematics calculation [50]. The forward kinematics reached an accuracy of 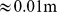, which corresponded to an oar angle accuracy higher than 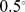. The direction from the oar lock 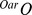 to the corresponding end-effector position 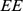 defined the orientations of the 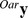 axis in both oar coordinate systems 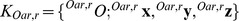 and 
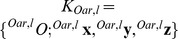
 (Figure 6). Consecutively, the horizontal oar angles 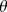 and the vertical oar angles 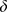 could be determined between the orientation of the initial oar coordinate systems 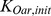 and the moved oar coordinate systems 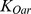. The third oar angle, the rotation of the oar 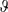 around its longitudinal axis 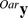, was measured by two wire potentiometers wound around each oar in parallel. Angle 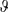 was defined to be 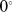, when the oar blade was parallel to the water surface and 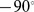, when the oar blade was vertical to the water surface. Both oars were fixed in oar locks that reduced the originally six degrees of freedom (DOFs) of each oar to three rotational DOFs. These three rotational DOFs were uniquely defined by the three oar angles 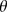, 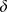, and 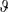, where 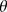 and 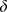 were actuated by the robot, while 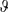 remained unactuated. In the scull simulator, the following movement variables 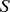 were recorded:

1. the two oar angles of both oars 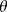 and 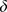 (resolution 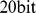 resulting in an accuracy higher than 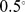),
2. the turning of the oar around its longitudinal axis of both oars 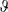 (accuracy higher than 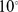),
3. the oar forces in three dimensional Cartesian space at the end-effectors of both oars (resolution of the force sensors at the end-effector in each drive train 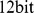 with a linearity of 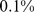 over a force range from 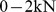),
4. the seat position 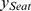 with a wire potentiometer measuring the distance between its mounting point on the boat hull and the back of the sliding seat (accuracy 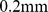),
5. and the distance 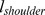 correlated with the rower's upper-body movement due to a wire potentiometer measuring the distance between its mounting point on the boat hull and a clavicle orthosis worn by the rower (accuracy 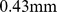).

**Figure 7:**
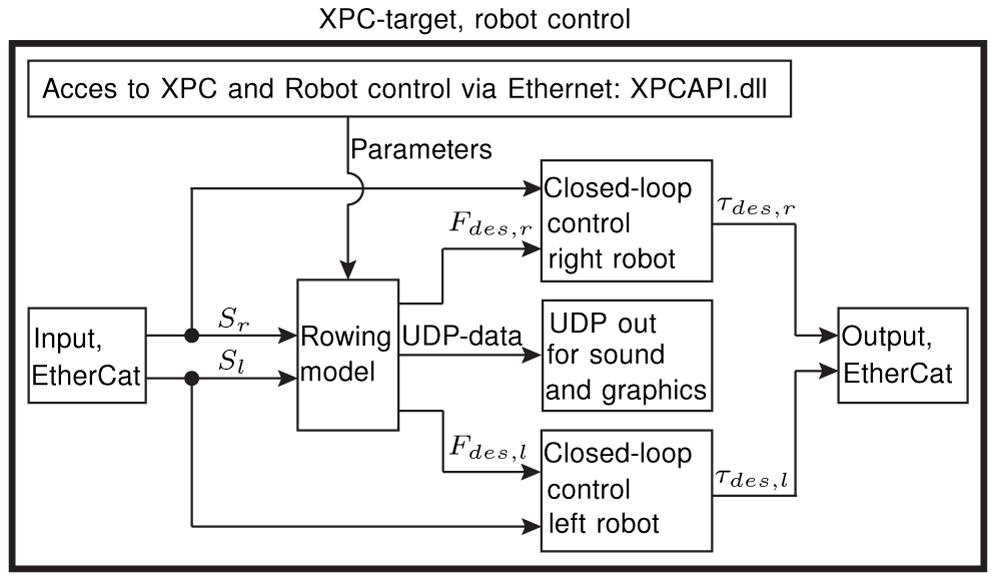
Schematic description of the *Matlab/Simulink*® model. This model is used to render sound and graphics as well as to control the two tendon-based parallel robots displaying haptic oar-water interactions.

The integral information on the movement variables was first transferred over *EtherCAT®* to a *Linux*™ PC and then via UDP to an XPC-target. A *Matlab/Simulink* model was used to control both robots simultaneously. In the robot control model, the recorded movement variables from the right robot 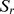 and the left robot 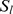 were processed by a rowing model. This rowing model determined the oar forces that should be rendered. A closed-loop force controller transformed these oar forces into desired motor torques 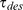 for each robot [43]. The signals for the desired torques were sent back to the *Linux*™ PC, which further communicated with the drives in the cabinet and all other sensors of the robot via *EtherCAT*® protocol. To synchronize all modalities in the virtual environment, the rowing model simultaneously determined the desired oar forces while generating UDP-data to trigger sound and graphics (Figure 7).

#### Rowing Model

For this study on transfer in sculling, a planar rowing model was developed that provided three DOFs for the rowing boat. The three DOFs were the movement in longitudinal- 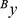 and lateral direction of the boat 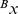, and the turning of the boat 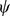 around its vertical axis 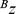 in the center of gravity (COG).

**Figure 8:**
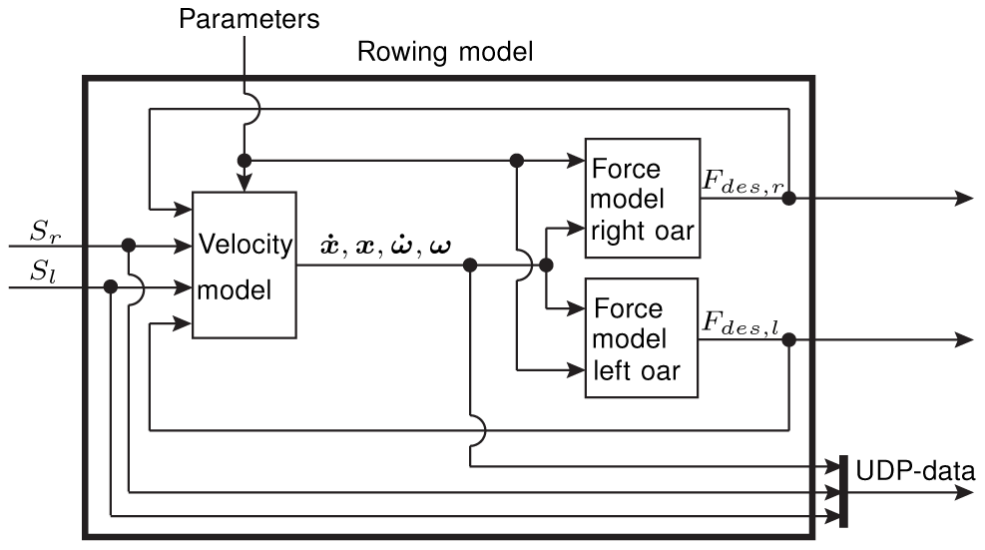
Data flow between the velocity model and the force models which constitute the rowing model. The rowing model gets the rower's movement as an input and drives the rendering of the acoustic, visual, and haptic scenario.

To determine the desired oar forces due to the interaction of the oars with the virtual water, all three oar angles (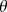, 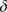, and 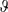) of both oars, the seat position 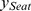, and the seat acceleration 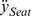 had to be included. These input data were provided by the measured signals from the right robot 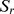 and the left robot 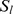. During each execution of the *Matlab/Simulink*® model (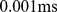), a multibody dynamics system (velocity model), comprising rower, boat, and the oars, was solved. This velocity model provided the boat's pose 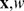 and velocity 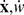, where 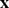 denoted the boat's position, 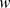 the boat's rotation, and 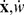 were their derivatives with respect to time. The boat's pose and velocity were needed for visual and auditory rendering. Moreover, the boat movement was used to determine the desired oar forces for each oar individually (force models for the right and left oar). Consequently, the resulting desired oar forces were used for haptic rendering with both robots. Finally, the desired oar forces served as input for the velocity model in the following iteration (Figure 8). A more detailed description of the velocity and the force models composing the rowing model can be found in the appendix (Appendix S1).

Due to the design of the scull simulator and the chosen functionalities of the implemented rowing model, the scull simulator has the following a priori known limitations:

1. the rower can experience oar-water forces due to a planar boat movement, however, the boat itself is stationary and the virtual reality scenario is moved around the rower in the stationary boat,
2. the dynamic behavior of the rowing model is reduced to a three DOF boat movement (a planar boat movement), while the remaining three DOFs are kept constant,
3. the center of gravity (COG) 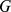 of the system rower-boat-oars is assumed to remain constant in the initial position of the rower's seat, therefore, turning of the boat will not exactly match turning of a real boat,
4. the boat-fixed coordinate system 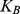 is defined in the COG 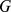 of the system rower-boat-oars at a predefined water level, therefore a changed rower mass does not have an effect on the boat drag, only on the inertia of the system rower-boat,
5. the boat only rotates around the axis 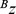 in 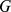, which defines the yaw angle of the boat 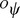, therefore, the rower's movement on the roll seat does not have an influence on the rotation axis of the boat,
6. the matrix of inertia of the system rower-boat in 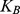 is in principal axis form and, therefore, movements in one direction cannot transfer energy into movements in another direction without the help of oar interactions,
7. environmental influences such as waves, other boats, etc. were not simulated.

### On-Water Measurements

**Figure 9:**
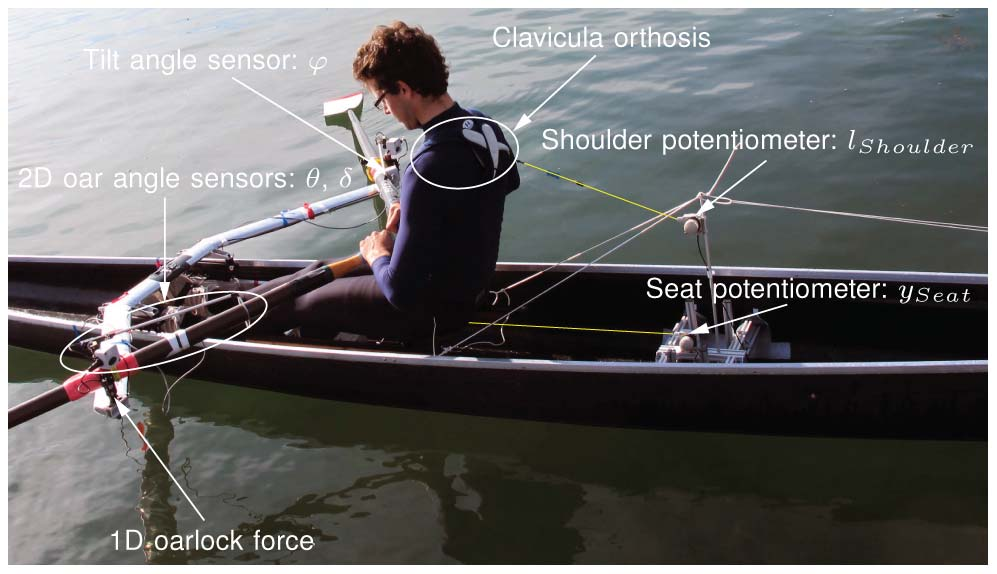
On-water measurement in a skiff for beginners. The skiff (Stämpfli Racing Boat AG, Switzerland) was equipped with a measurement system ( *BiorowTel*® version 3.5). This measurement system allowed for measurement of biomechanical performance measures in a similar way as in the simulator. The person of the photograph has given written informed consent, as outlined in the PLOS consent form, to publication of his photograph.

To assess transfer of simulator training by measurements on water, a skiff was equipped with a measurement system ( *BiorowTel*® version 3.5 from BioRow Ltd., Slough, UK). The following variables were measured at 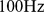 on water, matching those on the simulator (Figure 9):

1. the horizontal oar angles 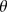 together with the vertical oar angles 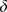 on both oars by 2D oar angle sensors (resolution 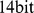 with a mean (standard deviation) in angular deviation of 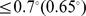),
2. the turning of both oars around their longitudinal axes 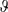 by binary tilt angle sensors (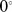, when the oar blade was parallel to the water surface and 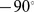, when the oar blade was vertical to the water surface),
3. the oar forces on both oars for pressure forces by 1D force sensors integrated in the oarlocks (resolution 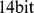 with a mean (standard deviation) in force deviation of 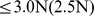),
4. the seat position 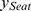 by a wire potentiometer measuring the distance between its mounting point on the boat hull and the back of the sliding seat (resolution 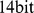 with a mean (standard deviation) in position deviation of 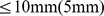),
5. and the distance 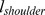 representing the rower's upper-body movement by a wire potentiometer measuring the distance between its mounting point on the boat hull and a clavicle orthosis worn by the rower (resolution 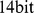 with a mean (standard deviation) in position deviation of 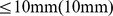).

For the measurements, a broad, full-carbon skiff for beginners provided by Stämpfli Racing Boat AG, Switzerland, was used. This boat provided increased stability against boat tilting, decreased the effect of waves. Thus, the intermediate rowers could focus on their coordination and oar handling technique.

### Participants

We were looking for participants who could improve their rowing performance within a short period of training and could row alone in a beginner's skiff without falling into water. Thus, the inclusion criteria were as follows:

1. recreational rower without competition experience,
2. participation in an intermediate's rowing course,
3. participation in a post-care rowing course,
4. less than two hours training per week,
5. healthy (no physical impairments or discomforts),
6. normal hearing, normal (or corrected to normal) vision,
7. age between 18 and 50 years.

Eight participants, four men and four women (mean age 35 years, 28 to 45 years), were included. The current study was conducted according to the regulation of the ethics commission of ETH Zurich. The corresponding ethics consent was obtained from the ethics commission of ETH Zurich: EK_2010-N-53. Furthermore, all participants signed a written consent form prior to the study. In addition, all participants were informed verbally about the study procedure, the risks, and their possibility to quit any time without a reason and without consequences. Moreover, all participants confirmed not to perform any rowing additional to the study. The participants were randomly assigned to one of two training groups, i.e. four to the on-water training group (*W*) and four to the simulator training group (*S*), i.e. the control group. Each group consisted of two women and two men.

### Experimental Protocol

The current study aimed at comparing the effectiveness of training in a realistic simulator to training on water. Consequently, the control group performed conventional rowing training on water (on-water group *W*) while the experimental group trained in the simulator (simulator group *S*). To keep the study close to real training conditions, a conventional training protocol for rowing was applied, i.e. a licensed human trainer guided the training sessions. The same licensed rowing trainer conducted the training sessions for all participants in order to eliminate the influence of different training methods. Furthermore, the rowing trainer did not prefer one training method to the other. In return, the human trainer's capacity, the scheduling and simultaneous availability of participants that fulfilled the criteria for the study, the availability of locations, boats, training-, and measurement equipment, as well as good environmental conditions e.g. little wind and good weather, limited the possible number of participants for the study to four participants per group.

**Figure 10:**
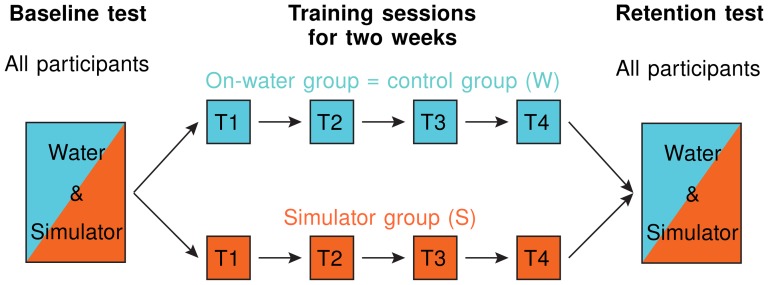
Study design for the two participant groups. Group 1 (on-water group = control group) trained on water (*cyan*) and group 2 (simulator group) in the simulator (*orange*). Before the training started, all participants underwent a baseline test under both conditions: in the simulator and on water. Then, both groups trained under their according condition for two weeks. During these two weeks, all participants trained four times under supervision of the same rowing trainer. Finally, both groups underwent a retention test similar to the baseline test.

After all participants had performed a baseline test on water and a baseline test in the simulator, all participants trained during four sessions in their respective group within a two week period. After the four training sessions, all participants were tested again under both conditions, i.e. on water and in the simulator (Figure 10). To evaluate the performance development of each participant, quantitative performance measures were derived from the variables recorded on water and on the simulator. In rowing, videos are commonly used to analyze the rowing technique. Therefore, also in this study, videos were taken of all participants during the baseline and retention tests on water. These videos were evaluated by a second, independent rowing trainer, who was blinded to the training conditions of the participants. Furthermore, the independent rowing trainer did not know if a video was taken during the baseline or during the retention test.

#### Baseline and Retention Tests

Each participant could adjust the foot stretcher individually. During the baseline tests, the foot stretcher settings were noted and applied again in the retention tests. The heights of the oar locks remained fixed for all participants throughout the whole study. Prior to the baseline and retention tests under both conditions, the participants could warm up for ten minutes. Then, they were asked to present their best rowing technique during three runs lasting three minutes each at a constant stroke rate of 20 strokes/min. The rowers got verbal instructions to increase or decrease their stroke rate as soon as they deviated more than 2 strokes/min. This correction of the stroke rate should keep the complexity level of the task constant. Overall, each participant performed 12 runs (four tests à three runs).

#### Training Goals

To be able to determine individual developments in rowing technique after four training sessions, training goals were defined together with both rowing trainers, i.e. the trainer, who carried out the training sessions and the one, who evaluated the videos. The training goals were defined based on two technical aspects that are commonly trained with intermediate rowers. The first training goal was the correct coordination of the body segments during drive phase, i.e. the coordination of legs, trunk, and arms. As a second training goal, the handling of the oars was selected due to its impact on efficient propulsive forces.

Since rowing techniques between the individual participants were expected to vary, the rowing trainer could emphasize technical aspects in the trainings according to the individual needs of each participant. During training, the trainer provided verbal feedback only on aspects that were predefined in the training goals. To keep track of the individual training emphasis, the rowing trainer documented the aspects that were trained individually. In this way, an individual evaluation of each participant's improvements was possible.

#### Training on Water

The on-water participants trained together on four different days during 70 min (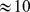 min warm-up and 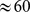 min training) on the lake. Training in small groups is the common practice in rowing training on water. Each participant rowed in a single skiff, which was of the same class as the instrumented skiff used for baseline and retention tests. The participants got individual verbal feedback from the rowing trainer, who accompanied the four rowers in a motor boat.

#### Training in Simulator

The four participants of the simulator training group trained individually, but with the same rowing trainer as the on-water group. To compensate for the advantage of an individual training compared to the on-water group and to compensate for the time needed for boat handling and preparations on water, the participants of the simulator group trained only for 50 min instead of 70 min, including 10 min of warm-up. For safety reasons, the trainer provided verbal feedback from outside the CAVE. However, he could observe the participant from outside the CAVE from different sides and also by switching between starboard and stern view of a real-time video stream on a TV-screen.

### Biomechanical Performance Measures

To enable a quantitative comparison between the development in rowing performance on water and in the simulator, ten biomechanical performance measures (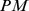) were defined together with the rowing trainers in accordance with literature [47], [51], [52]. Furthermore, these 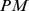 should cover the key measures that the rowing trainer, who performed the trainings, intended to use to instruct the participants. At the same time, the 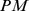 should also be the key measures that the independent rowing trainer, who performed the blinded video evaluation of the baseline and retention tests on water, would need to evaluate the individual development of each participant. The 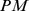 covered four categories: the two technical aspects, i.e. “oar handling skills” and “coordination of body segments” and two general categories, i.e. “oar angles” and “power”. “Oar handling skills”, were represented by the following sub aspects: (i) “catch slip”, (ii) “depth of the blade immersion”, and (iii) “striking out before catch”. “Coordination of body segments” could be subdivided into: (i) “temporal overlap in the movement of legs and trunk during drive phase” and (ii) “overlap in the movement of trunk and arms during drive phase”. The “oar angles” consisted of (i) the “catch angle”, (ii) the “release angle”, (iii) and the “stroke length”. The “power” was subdivided into (i) “maximal power at the oar handle” and (ii) “mean power at the oar handle”. All performance measures for the oars were derived from measurements on the left-hand oar, i.e. starboard oar in the simulator and on water. The data for the right oar was not used due to a deficiency of the measurement of the vertical oar angle 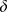 in the right-hand oar (port side oar) during all retention tests on water. The turning of the oars around their longitudinal axis 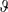 was not considered in the performance measures. A detailed description of all ten biomechanical performance measures can be found further down in this chapter.

Only one out of three runs from baseline tests (one from on-water and one from the simulator condition) and one out of three runs from retention tests (one from on-water and one from the simulator condition) were considered to evaluate a participant. The runs were chosen on their consistency in performance measures. These performance measures were: direct catch, depth of the blade immersion, striking out before catch, overlap of leg and trunk movement, overlap of trunk and arm movement, catch angle, release angle, and stroke length. Consistency of a run was evaluated by the sum of the coefficients of variation 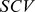 for these eight performance measures:
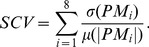
(1)Where 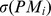 denotes the standard deviation of the performance measure with index 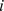, and 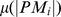 is the mean of the absolute values of the 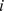-th performance measure. Hereby, high consistency was defined by low values in the 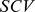. The most consistent runs were used to evaluate the individual development in performance from baseline to retention 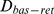 for each participant. To quantify the development, the difference between the performance measures during baseline 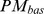 and retention tests 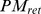 was set in relation to the pooled standard deviation 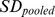:
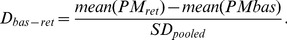
(2)The pooled standard deviation 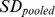 is commonly used to express the effect size of a variable. It considers the performance measure of standard deviations of baseline- 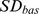 and retention tests 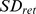. Furthermore, the pooled standard 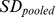 considers the number of rowing cycles in baseline 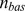 and retention 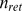 that were used to calculate the standard deviations 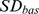 and 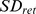:
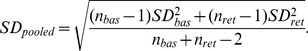
(3)Due to the relatively small group size, a change in a performance measure was considered as significant if the change was at least in the order of one pooled standard deviation.

### Definition of the Ten Biomechanical Performance Measures

#### 1. Catch Slip

The more direct the blade is moved into the water after the catch 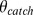, the earlier propulsive forces can be applied to propel the boat. As a performance measure for a direct catch, the opposite is taken: the loss of stroke length, i.e. the “catch slip” 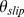. The “catch slip” is characterized as the difference between the catch angle 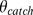 and the horizontal oar angle, when the blade is fully immersed in the water 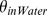 [51], [52]:
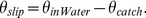
(4)Thus, small values for catch slip are desirable to increase the effectiveness of a stroke.

#### 2. Depth of the Blade

When the oar blade is immersed too deeply into the water during drive phase, the release gets more demanding. An improper release leads to boat perturbations, a loss of propulsive forces, or even to breaking forces. To determine the depth of the blade 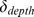, the difference between the water level 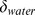 and the deepest point of the blade during the drive phase 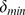 was calculated:
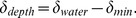
(5)Thus, in the optimal case, the depth of the oar blade corresponds to the change in the vertical oar angle 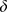 that is needed to completely immerse a previously floating oar just below the water surface.

#### 3. Striking out Before Catch

The striking out movement is characterized by a rising of the vertical oar angle 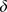 at the end of the recovery phase, i.e. in the preparation of the catch. Striking out can be caused by pushing the hands down extensively or by buckling the trunk. Striking out can disturb the stability of the boat or hinder a direct catch. The performance measure for striking out before catch 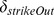 was calculated by the difference between the maximal vertical oar angle 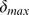 and the vertical oar angle when the horizontal oar angle was zero before the next catch 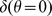:
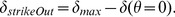
(6)For a good technique, no striking out movement should be detected.

#### 4. Overlap Legs-Trunk

The correct coordination of the main body segments is the key to a good rowing performance. Wrong coordination between legs, trunk, and arms can lead to early exhaustion of the rower, lower power transferred to the oars, shortened stroke length, or back pain. Thus, legs, trunk, and arms should be moved sequentially to transfer forces optimally to the oar [52]. The performance measure “overlap legs-trunk” 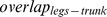 quantifies the overlap in the sequential movement of legs and trunk in percent of a rowing cycle 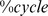. The overlap between the movements of two body parts should be as small as possible. The “overlap legs-trunk” was calculated in the following way:
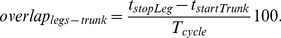
(7)Here, 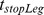 denotes the point in time when the legs stopped to move, 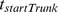 was the point in time when the trunk started to move, and 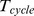 was the duration of the entire rowing cycle. The movement of legs, trunk, and arms depended on the rower's anthropometry.

#### 5. Overlap Trunk-Arms

The performance measure “overlap trunk-arms” 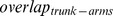 quantifies the overlap in the sequential movement of trunk and arms in percent of a rowing cycle 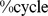, similar to the “overlap legs-trunk”:
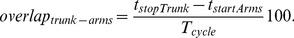
(8)In this equation, 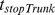 denotes the point in time when the trunk stopped to move, and 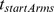 is the point in time when the arms started to move. Ideally, the overlap between trunk and arms should be as small as possible.

#### 6. Catch Angle

The start/end of a rowing cycle, i.e. the start of the drive-, or the end of the recovery phase, is characterized by the catch angle 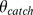. Trainers usually instruct the rowers to decrease their catch angle, which increases their stroke length and consequently maximizes their propulsion. Therefore, in this paper, the catch angle was defined as the minimal horizontal oar angle 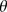 of each stroke.

#### 7. Release Angle

The end of the drive-, or the start of the recovery phase, is characterized by the release angle 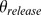. Analogously to the catch angle 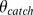, the release angle was defined as the maximal horizontal oar angle 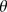 in each rowing cycle. In order to maximize the propulsion for each stroke through increase of the stroke length, the release angle 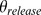 should be maximized.

#### 8. Stroke Length

The stroke length is the range of motion (ROM) in horizontal direction of the oar from the catch to the release. Thus, the stoke length was obtained in the following way:
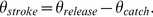
(9)The stroke length should be matched to the rower's body size. Given the individual limitations through body size, the stroke length should be maximized to obtain maximal propulsion, while other performance measures should not be negatively affected.

#### 9. Maximal Handle Power

The maximal handle power 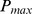 was calculated in the following way:
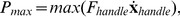
(10)where 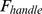 indicates the force at the oar handle parallel to 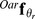 and 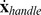 is the velocity of the handle in force direction obtained by 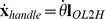. The maximal handle power 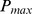 can be used to indicate wrong coordination of the body segments. High maximal power is desirable, since it contributes directly to propulsion of the boat.

#### 10. Average Handle Power

The average handle power 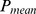 was calculated in the following way:
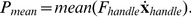
(11)High values for 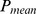 are necessary to reach high boat velocities.

### Video Assessment

In addition to the assessment by 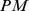, videos of all on-water tests were taken. The performance of all participants was rated by a second, independent rowing trainer, who was blinded to the group assignment of the participants. Also, he did not know if a video showed a baseline or a retention test. Seven technical aspects were rated according to the degree of error occurrence. The rating scale ranged from 0 ( = no occurrence) to 4 ( = very strong occurrence). All seven technical aspects that were rated corresponded to one out of the biomechanical performance measures. The stroke length, the mean power, and the maximal power were difficult to rate by video assessment and were therefore not considered by the independent rowing trainer. A comparison between the 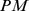 and their corresponding video rating was performed to test the validity of the 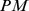 for quantitative assessment of rowing performance on water.

In addition to the technical aspects quantified by 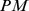, the independent rowing trainer rated general aspects such as the rowing rhythm, dynamics of the technique, and provided a general impression. The general aspects were rated in a scale from 1 (very bad) to 6 (very good). These general aspects were intended to document the general development of each participant.

### Questionnaire

All participants had to fill out a questionnaire including 21 questions (six categories). In the following, these six categories are explained through a typical question for each category:

1. “Involvement/control”: How deeply involved into the virtual environment did you feel? How well could you control the oar in the virtual world?
2. “Naturalism”: How natural did the boat movement in the simulator appear to you?
3. “Advantage simulator”: Was it helpful for you that you were able to fully concentrate on the rowing technique in the simulator without environmental disturbances?
4. “Interface quality”: How natural did the haptic interaction with the water feel?
5. “Engagement trainer”: How well did the rowing trainer supervise you during the trainings?
6. “Personal profit”: Could you personally profit from this rowing course?

The rating score ranged from 1 (worst rating) to 7 (best rating).

Questions on category three were only asked to participants in the simulator group. The questions of category 1 to 4 were adapted from [53]. In addition, participants were asked if they had performed extra rowing training beyond the study.

## Results

For the evaluation of the current study, the most consistent runs from the baseline and retention tests on water and in the simulator were chosen. Highest consistency in the data of the three runs per test was defined as the lowest value in the sums of coefficients of variation of the variables for the categories “oar handling skills”, “body segments coordination”, and “oar angles”. In general, the values of the coefficients of variation were lower during the tests on the simulator than during the tests on water. The individual consistency of each participant was assessed by mean values of the sums of coefficients of variation over all tests. Here, participant “W2” indicated the highest consistency of all, followed by participants “S1” and “S4” (Table 1).

**Table 1:** Sums of coefficients of variation for all runs, tests, and participants.

Test condition	Run	W1	W2	W3	W4	S1	S2	S3	S4	Mean: runs 1–3
Water Baseline	Run1	***0.12***	**0.11**	**0.22**	**0.30**	**0.16**	**0.21**	**0.20**	**0.14**	
Water Baseline	Run2	0.17	***0.06***	***0.14***	**0.22**	***0.07***	***0.12***	***0.09***	***0.10***	**0.16**
Water Baseline	Run3	0.15	0.18	0.18	***0.22***	**0.11**	**0.14**	**0.12**	**0.17**	
Water Retention	Run1	0.31	0.10	0.20	0.14	0.14	***0.14***	**0.16**	**0.15**	
Water Retention	Run2	***0.28***	***0.08***	**0.17**	***0.14***	***0.13***	**0.16**	***0.11***	**0.16**	**0.17**
Water Retention	Run3	0.35	0.08	***0.16***	**0.18**	**0.14**	**0.20**	**0.14**	***0.14***	
Simulator Baseline	Run1	***0.09***	**0.07**	**0.22**	*	**0.13**	**0.22**	**0.15**	***0.10***	
Simulator Baseline	Run2	0.11	***0.05***	**0.23**	***0.16***	**0.17**	**0.15**	***0.14***	**0.12**	**0.14**
Simulator Baseline	Run3	0.09	0.06	***0.22***	*	***0.12***	***0.14***	**0.15**	**0.10**	
Simulator Retention	Run1	***0.19***	**0.06**	**0.14**	**0.22**	***0.07***	**0.12**	**0.09**	***0.10***	
Simulator Retention	Run2	0.27	***0.06***	**0.13**	***0.13***	**0.09**	**0.16**	**0.09**	**0.10**	**0.12**
Simulator Retention	Run3	0.22	0.06	***0.13***	**0.16**	**0.10**	***0.12***	***0.09***	**0.11**	
**Mean**		0.20	0.08	0.18	0.19	0.12	0.16	0.13	0.12	

***Italic bold font***: indicates the runs with the lowest coefficients of variation for each test and participant. These runs were taken for the evaluation of the study.

* Run could not be used.

During the baseline and retention tests, all participants were asked to row at 20 strokes/min to ensure comparable conditions throughout all tests. For the selected runs, all participants maintained the desired stroke rate without exceeding the desired limits of 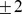 strokes/min during both tests in the simulator, except “S4” in the baseline test. The deviations in the mean values of the individual stroke rates from the desired stroke rate confirm that it was more challenging for the participants to maintain the desired stroke rate on water than on the simulator (Table 2).

**Table 2:** Stroke rates development.

Stroke rate: water	W1	W2	W3	W4	S1	S2	S3	S4	Mean
*Mean baseline*	23.5	21.1	15.9	20.7	17.0	19.9	18.1	17.5	19.2
*SD baseline*	0.4	0.4	1.2	0.7	0.5	0.5	0.7	0.4	0.6
*Mean retention*	21.0	21.6	19.2	20.8	20.1	21.6	21.8	21.1	20.9
*SD retention*	1.0	0.4	0.6	0.5	0.4	0.7	0.4	0.6	0.58
*Difference*	−2.4	0.5	3.3	0.0	3.2	1.7	3.7	3.6	1.7

Stroke rate: simulator	W1	W2	W3	W4	S1	S2	S3	S4	Mean
*Mean baseline*	20.4	20.6	19.5	19.4	19.6	19.8	21.0	18.5	19.9
*SD baseline*	0.7	0.4	0.6	0.6	0.6	0.7	0.5	0.8	0.6
*Mean retention*	19.5	20.7	20.8	20.6	20.1	19.4	20.3	21.0	20.3
*SD retention*	0.6	0.3	0.7	0.4	0.8	0.9	0.8	0.5	0.6
*Difference*	−0.9	0.1	1.3	1.2	0.4	−0.3	−0.7	2.5	0.4

Development of the stroke rates for all participants during baseline and retention test, on water and in the simulator. Desired stroke rate was 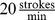.

To quantify the development of all participants, the differences between the biomechanical performance measures during baseline and retention tests for on-water and on-simulator tests were calculated (Table 3). Through comparison of the individual developments of the participants, it was found that all participants developed differently. The increase/decrease in performance between training groups and test conditions was quantified by summing up the “sums of development” for each training group and test condition. On the simulator, the on-water group improved in 14 biomechanical performance measures, stayed indifferent in 21, and degraded in 5, while the simulator group improved in 17, stayed indifferent in 17 and degraded in 6. On water, the on-water group improved in 13 biomechanical performance measures, stayed indifferent in 20, and degraded in 7, while the simulator group improved in 6, stayed indifferent in 20, and degraded in 14. In terms of the qualitative scale of the video analysis, the on-water group improved in 12 technical aspects on water, stayed indifferent in 13, and degraded in 3, while the simulator group showed an increase in 7, stayed indifferent in 15 and decreased in 6.

**Table 3:** Development of biomechanical performance measures in all participants from baseline to retention.

	W1			W2			W3			W4			S1			S2			S3			S4		
	S	W	V	S	W	V	S	W	V	S	W	V	S	W	V	S	W	V	S	W	V	S	W	V
**Oar handling skills**																								
*Catch slip*	***1.11***	***−0.27***	***0***	***0.04***	***0.50***	***0***	***0.31***	***−1.14***	***0***	***−0.27***	***−0.43***	***2***	***−1.36***	***−0.35***	***1***	***3.17***	***−0.19***	***2***	***−1.79***	***1.85***	***0***	***6.21***	***−0.90***	***−1***
*Depth of blade*	***0.11***	***1.40***	***1***	0.19	−0.57	1	***0.00***	***−0.13***	***1***	***1.83***	***1.55***	***1***	***0.65***	***−0.30***	***1***	***1.22***	***1.31***	***−1***	***0.65***	***−1.58***	***0***	−1.28	−1.66	0
*Striking out before catch*	−1.39	−1.92	0	0.54	−1.04	0	0.82	−0.99	−1	−1.04	0.04	1	2.81	1.51	2	***1.13***	***0.99***	***0***	***1.35***	***−1.09***	***0***	−0.01	−3.74	−1
**Body segments coordination**																								
*Overlap legs-trunk*	1.20	−1.31	−2	***−0.65***	***2.59***	***1***	1.88	1.81	0	***2.78***	***0.22***	***0***	***1.45***	***−0.11***	***0***	0.36	−0.52	1	***0.01***	***−0.80***	***1***	1.53	0.66	1
*Overlap trunk-arms*	1.38	−0.49	1	−0.89	0.12	0	1.61	0.65	0	***2.06***	***0.28***	***0***	***1.34***	***−1.13***	***0***	−0.65	−0.59	0	***0.23***	***−0.41***	***0***	0.85	−0.50	0
**Oar angles**																								
*Catch angle*	***1.91***	***4.06***	***0***	0.54	4.86	1	0.87	−2.46	−1	***0.88***	***−3.28***	***2***	***2.77***	***−2.25***	***−1***	***0.63***	***1.02***	***0***	***9.08***	***0.43***	***−1***	***1.35***	***−2.17***	***0***
*Release angle*	***−1.74***	***0.61***	***1***	***0.84***	***2.32***	***2***	***−0.05***	***0.93***	***0***	***2.22***	***0.61***	***0***	***−0.73***	***0.10***	***0***	***−3.30***	***−2.35***	***0***	−0.76	−1.60	−2	−2.03	−1.30	0
*Stroke length*	0.71	2.42		0.95	4.25		0.43	0.07		1.73	−1.03		0.75	−0.72		−1.63	−1.12		6.97	−0.71		−0.73	−2.07	
**Power**																								
*Max. handle power*	0.96	0.93		−2.89	1.84		1.71	2.17		1.30	−0.75		0.74	0.30		−0.68	−2.17		1.94	1.69		−0.34	−0.64	
*Mean handle power*	0.82	0.72		−1.53	1.53		2.26	2.71		0.89	−0.85		1.17	−0.08		−0.55	−2.13		1.83	2.77		1.50	−0.29	
**Sum of developments**	4/2	3/2	3/1	0/2	6/1	4/0	4/0	3/2	1/2	6/1	1/2	4/0	5/1	1/2	3/1	3/2	2/4	2/1	5/1	3/3	1/2	4/2	0/5	1/2

Simulator (S) and on-water tests (W) were quantified in terms of pooled standard deviation (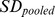). A significant improvement/degradation in a technical aspect was indicated by values larger/smaller than one, respectively. On-water performance was also qualitatively evaluated by video assessment (V) (−4 to 4 in steps of 1: negative/positive values indicated a performance decrease/increase). ***Italic bold font***: this particular aspect was instructed during training. Empty cells: no video assessment was performed for this technical aspect.

**Figure 11:**
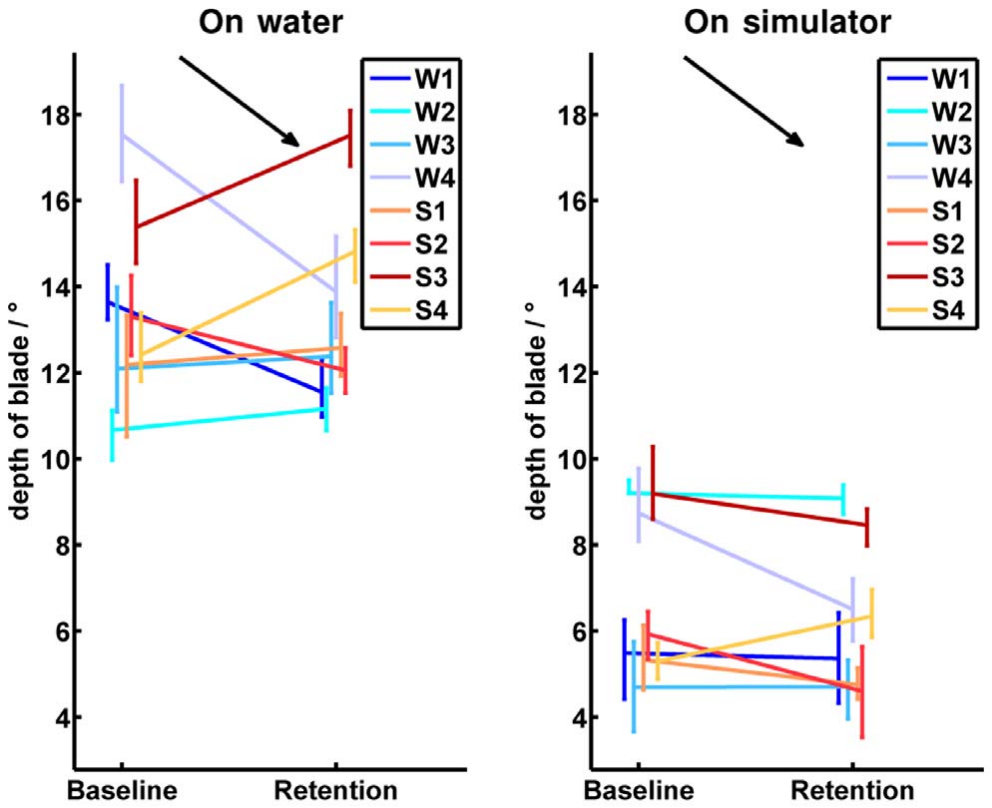
Absolute values of the depth of blade immersion. The left half image shows the development of all participants from baseline to retention tests on water, while the right half image illustrates the baseline and retention measurements on the simulator. Participants of the on-water group are indicated by bluish colors and abbreviated by 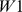 to 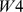 in the legend. Participants of the simulator group are indicated by reddish colors and abbreviated by 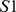 to 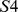 in the legend. The vertical bars in the baseline and retention tests cover all values performed by the corresponding participant. The measured values in the baseline and the retention tests are connected in the mean values for each participant. The black arrow indicates the direction of the desired development from baseline to retention in the corresponding test environment. In general, the oars should be just fully immersed. In the simulator, the oars were completely immersed at a depth of blade of 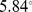. On water, the depth of blade depended on the rower's weight, balance, and the waves. However, due to differences in rigging between the measurements on water and on the simulator, the oars were completely immersed on water at a depth of blade around 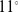.

A characteristic development of the “oar handling skills” could be found in the “depth of blade”. All participants showed an offset in depth of the oar blade in the simulator compared to on-water rowing. The smallest offset of less than three degrees was found in subject “W3”, while all other subjects yielded offsets larger than five degrees. A group dependent development could not be found. However, participant “W4” improved significantly under both conditions (Figure 11).

**Figure 12:**
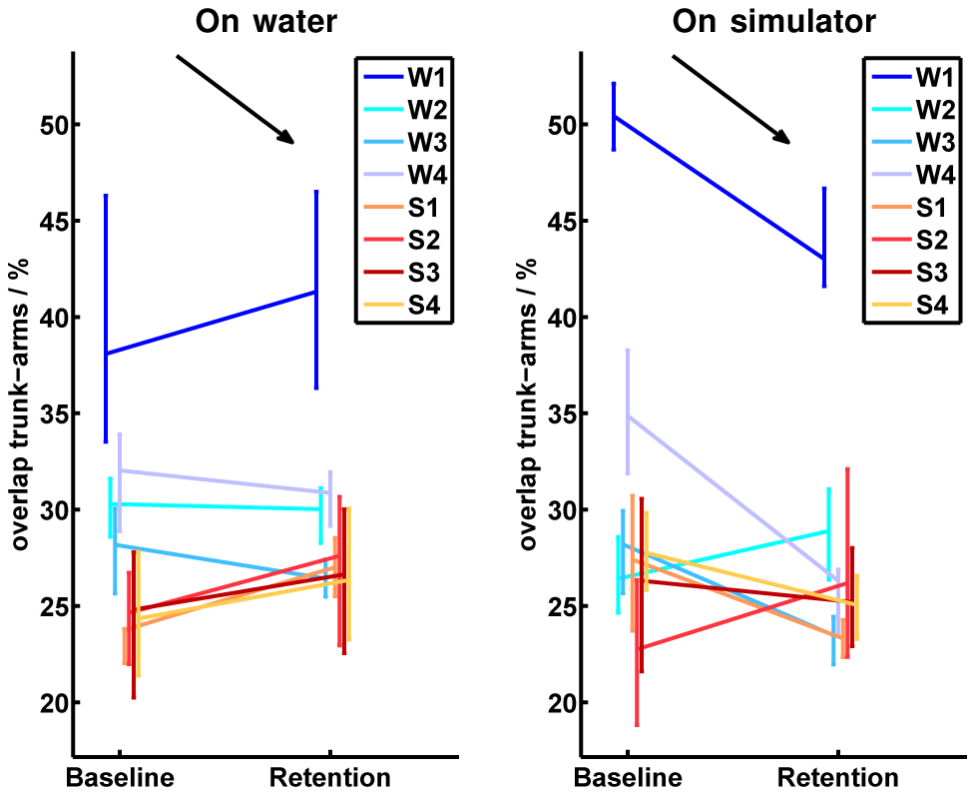
Absolute values of the overlap between trunk and arms. The left half image shows the development of all participants from baseline to retention tests on water, while the right half image illustrates the baseline and retention measurements on the simulator. Participants of the on-water group are indicated by bluish colors and abbreviated by 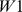 to 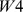 in the legend. Participants of the simulator group are indicated by reddish colors and abbreviated by 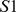 to 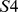 in the legend. The vertical bars in the baseline and retention tests cover all values performed by the corresponding participant. The measured values in the baseline and the retention tests are connected in the mean values for each participant. The black arrow indicates the direction of the desired development from baseline to retention in the corresponding test environment.

In terms of the “body segments coordination”, all participants generally performed coordinative movements in a similar range under both conditions. The only exception was subject “W1”, who showed difficulties in coordination from the beginning on. However, similar to participant “W4”, “W1” could significantly improve her coordination on the simulator from baseline to retention. A clear trend in the development of the different training groups was not found (Figure 12 exemplifies the body segments coordination by the overlap between trunk and arms during pulling phase).

**Figure 13:**
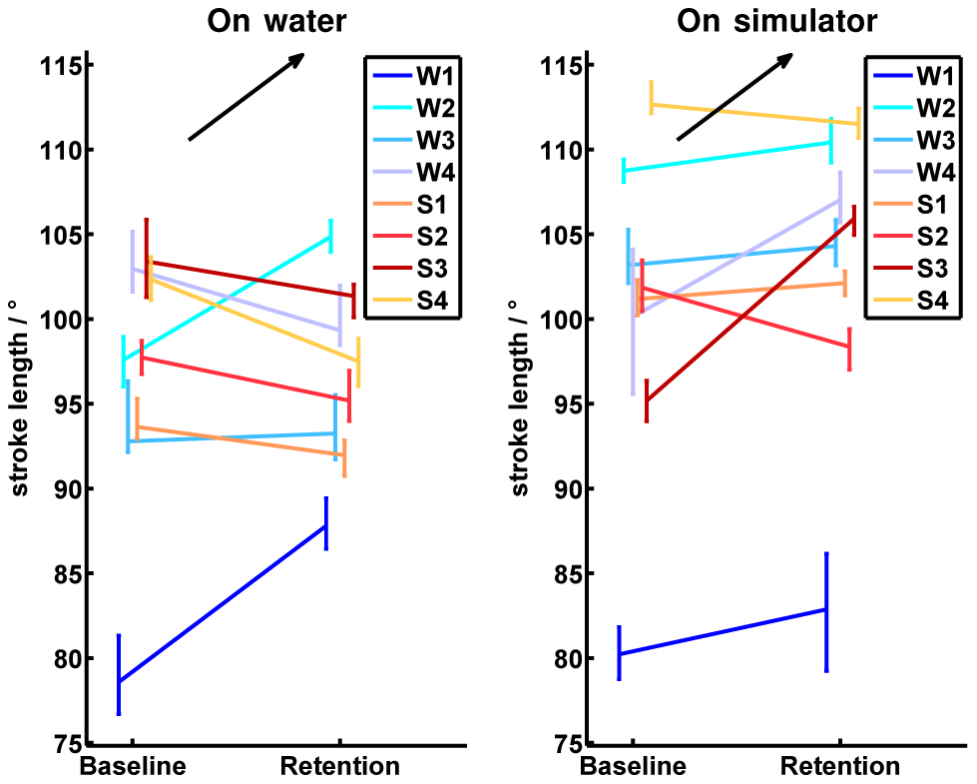
Absolute values of the stroke length. The left half image shows the development of all participants from baseline to retention tests on water, while the right half image illustrates the baseline and retention measurements on the simulator. Participants of the on-water group are indicated by bluish colors and abbreviated by 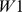 to 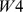 in the legend. Participants of the simulator group are indicated by reddish colors and abbreviated by 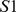 to 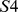 in the legend. The vertical bars in the baseline and retention tests cover all values performed by the corresponding participant. The measured values in the baseline and the retention tests are connected in the mean values for each participant. The black arrow indicates the direction of the desired development from baseline to retention in the corresponding test environment.

Development of the “oar angles” is exemplified on the “stroke length”. In the simulator, the participants reached an increased stroke length by 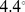 on average compared to on-water. While the simulator group decreased in stroke length from 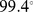 to 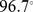 on average on water, the on-water group increased from 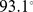 to 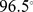 on average. A significant improvement from baseline to retention tests were found in participants “W1” and “W2” on water (Figure 13).

**Figure 14:**
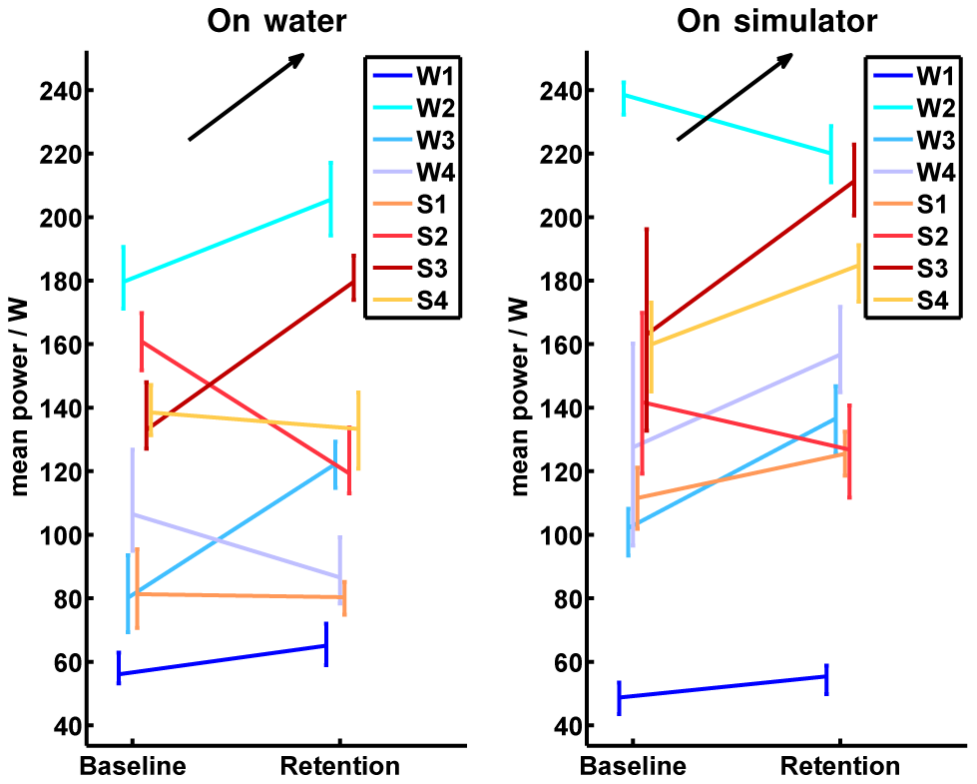
Absolute values of the mean power. The left half image shows the development of all participants from baseline to retention tests on water, while the right half image illustrates the baseline and retention measurements on the simulator. Participants of the on-water group are indicated by bluish colors and abbreviated by 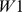 to 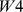 in the legend. Participants of the simulator group are indicated by reddish colors and abbreviated by 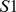 to 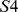 in the legend. The vertical bars in the baseline and retention tests cover all values performed by the corresponding participant. The measured values in the baseline and the retention tests are connected in the mean values for each participant. The black arrow indicates the direction of the desired development from baseline to retention in the corresponding test environment.

The development of the “power” is exemplified by the “mean power”. On average, the mean power produced by all subjects was 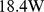 higher on the simulator than on water. Furthermore, the subjects could increase the produced power by 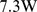 on average from baseline to retention on water, and by 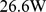 on the simulator. In general, no differences between groups were found and the rankings of the participants according to mean power produced in the simulator and on water were similar (Figure 14).

An evaluation of the general development in rowing performance by the independent rowing trainer revealed an increase in performance in at least one out of three general performance measures in all participants, but in participant “S4” (Tab. 4). Note that the initial skills of the participants from the simulator group were on average one point superior to the initial skills of the on-water group. After retention tests, the simulator group showed still a slight advantage in performance (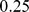 points on average) compared to the on-water group.

**Table 4:** Video assessment of the rower's general development (D) from baseline (B) to retention (R).

	W1			W2			W3			W4			S1			S2			S3			S4		
	B	R	D	B	R	D	B	R	D	B	R	D	B	R	D	B	R	D	B	R	D	B	R	D
*Dynamics*	2	3	1	4	55	1.5	3	5	2	5	5	0	5	5	0	4.5	5	0.5	5	5	0	5	5	0
*Rhythm*	3	4	1	4.5	55	1	3	5	2	4	4.5	0.5	3	4.5	1.5	4.5	4.5	0	4	4	0	5.5	5	−0.5
*Overall impression*	3	4	1	4.5	6	1.5	3.5	5	1.5	5	5	0	5.5	6	0.5	5	5.5	0.5	4.5	5	0.5	5	4.5	−0.5

The independent rowing coach evaluated the aspects: dynamics (flow of motion, forward motion), rhythm (ratio between drive phase and recovery phase), and the overall impression. The rating ranged from 1 to 6 in steps of 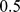, where 1/6 indicated a bad/excellent rating.

**Figure 15:**
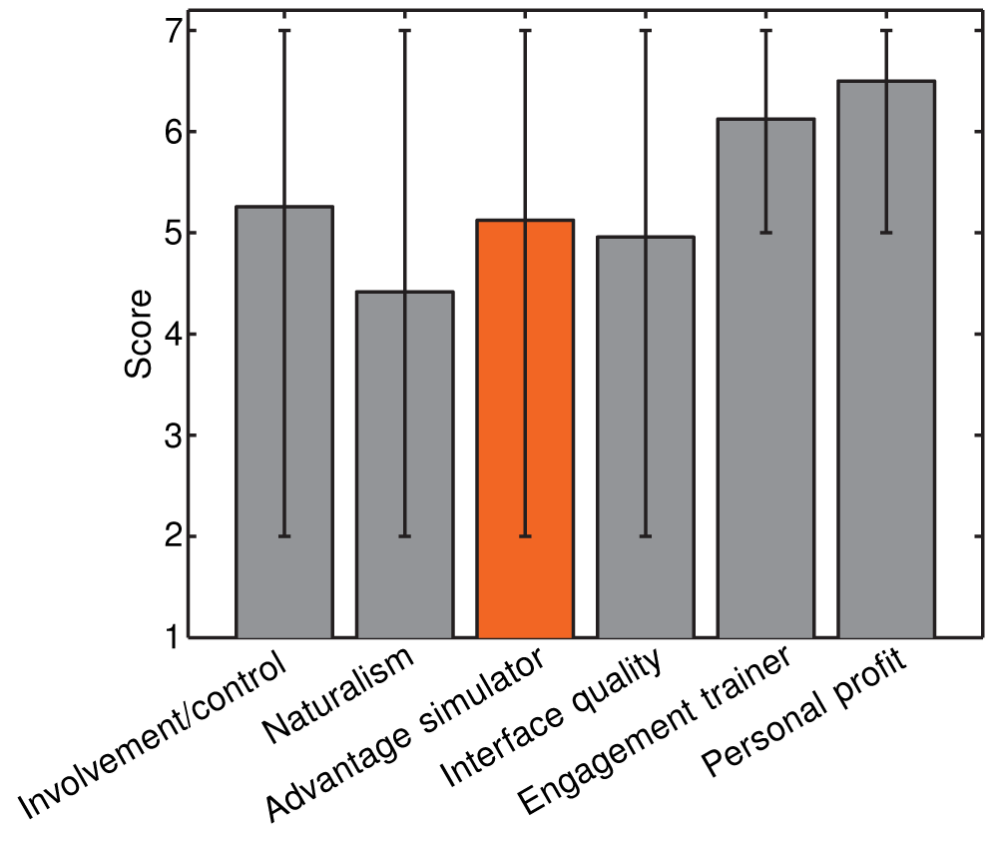
Results of the participants questionnaire on different categories of questions. *Grey* and *orange* bars indicate the mean value over all questions for a certain category. Bars in *orange* indicate questions that were only asked in the simulator group. The small bars indicate the range of scores (minimal to maximal).

The questionnaire rating score ranged from 1 (worst rating) to 7 (best rating). The average rating of “involvement/control”, “naturalism”, “advantage simulator”, and “interface quality” of the simulator was 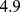. All participants had a very good impression of the trainer's engagement (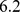) and could personally benefit from the study (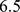). Furthermore, the majority of the simulator group saw an advantage in technique training in the simulator compared to training on-water (Figure 15).

Finally, the participants were asked if they had respected the agreement not to perform any rowing-specific training during the period of the study. All participants but one confirmed. Participant “S4” admitted to have once trained sweep rowing in an eight and to have performed extra training on a *Concept2*® ergometer.

## Discussion

Simulator training is effective, when the skills learnt on the simulator transfer to the real task [34]. To feature transfer, the simulator has to represent the key features of the real task well enough, since key features are crucial during development of a movement plan [54]. Previous transfer studies focused on tasks involving hand-eye-coordination and provided augmented feedback or modifications of physical parameters in the simulator [35], [37]. In contrast, the current transfer study did not only include audiovisual rendering but also realistic haptic rendering of interactions with a virtual environment. The level of realism of these multimodal interactions in our simulator was hypothesized to provide all important key features to enable a transfer of learnt skills to the real task, i.e. on-water rowing. Augmented feedback, modification of physical parameters, as well as manipulation of the training protocol were omitted explicitly since we were solely interested in the basic skill gain, and the participants' acceptance of realistic simulator training. We hypothesized that the rowers of the on-water training group could improve their individual rowing skills on water. To a similar extent, the simulator training group was expected to improve their rowing skills on the simulator, and, importantly, was expected to transfer these skills to rowing on water.

### Movement Consistency

Consistency is known as an indicator for expert performance [55]. The results of this study support this indication: Participants “W2”/;“W1”, who showed the least/highest values in the sums of coefficients of variation also got the best/worst ratings in the assessment of general aspects. Generally, the ranking of the participants according to “overall impression in the development” was similar to the participants' ranking according to consistency (Table 4: row: “overall impression”, columns “D”; compared to Table 1: last row).

The participants' consistency during the baseline tests on water was by 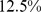 lower than on the simulator. During the retention tests on water, the participants' consistency was even 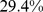 lower than on the simulator (Table 1: last column). Probably, external factors such as waves, wind, and other boats, had an impact on the consistency of on-water rowing. Such environmental influences were absent in the simulator which might be supportive particularly in early learning phases.

### Individual Development

All participants were selected upon rather strict criteria; nevertheless slight differences in initial skill-level between participants had to be expected. Furthermore, the participants were expected to show individual deficits in different 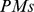 with respect to the predefined training goals. Therefore, the rowing trainer was allowed to provide individual verbal feedback with respect to deficient 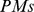. The 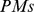 that were explicitly trained in this way were indicated by *italic font* in Table 3. At a close look, only participants 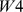 and 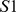 got instructions concerning the same 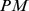. All other participants trained other combinations of 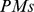. This inconsistency originated from the trainer's intuition to satisfy the participants' need for individual training, e.g. to train the most prominent deficits first. The participants' need for individual training is especially supported by the fact that the participants exhibited large differences in their initial skill level (Table 4: “overall impression” in the baseline tests). These differences in initial skill level combined with individual training might explain to a large part why all participants developed differently in the 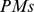 (Table 3). Although learning is an individual process that depends on many factors, all participants could improve in at least one general aspect from baseline to retention on water according to the video assessment. The only exception was participant “S4”, who did not follow the training protocol and performed extra rowing trainings in different conditions besides this study (Table 4). This general gain in performance indicates that the applied training protocol from conventional rowing training with a human trainer was adequate for the current study.

### Biomechanical Performance Measures Compared to Video Assessment

Considering the total sum of 17 improved and 6 worsened 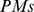, the simulator group principally improved their skills on the simulator. Similarly, the on-water training group improved in 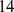 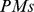 and degraded in 5 on the simulator. Thus, the on-water group could transfer the learned skills trained on water to the simulator.

On water, the on-water group increased performance in 13 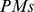, whereas only 7 developments showed a degradation. This development was also confirmed by the video assessment that indicated an improvement of 12 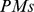, while only 3 developments degraded. The simulator group showed a total of 6 improvements in 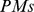, while 14 parameters indicated a decrease in performance development. Thus, more 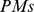 were found to be degraded than improved. However, this development was not confirmed by the video assessment of the 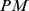 through the independent, blinded rowing trainer. The video assessment of the 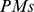 revealed a total of 7 improvements and 6 degradations (Table 3). In our experience, the following points could be discussed to explain the discrepancy between the 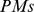 and the video assessment for the development:

1. The evaluation of the 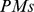 was based on all strokes, i.e. also on irregular or externally disturbed strokes. Thus, characteristic behavior of the rower might be weakened by averaging over all cycles. In contrast, the independent human trainer, who evaluated the videos, might have focused on characteristic performance in the strokes he judged as regular. Furthermore, a human trainer is able to rate performance particularities in relation to an overall impression of an athlete. To improve the biomechanical analysis, machine learning techniques could be applied to train algorithms that can classify and rate data in relation to the overall impression of an athlete's performance. This has been tried already in rowing for one specific rowing error [56].
2. Due to the influence of weather and waves, the participants might have been forced to row in a different way in the baseline test than in the retention test. While the independent rowing trainer could take the influence of the environmental conditions into account, the 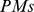 did not adapt to changed conditions.
3. Although the 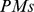 were selected based on literature and in discussions with both trainers, the 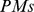 were not sufficient to capture the individual performance especially of more skilled participants. This conclusion is supported by the documented instructions given by the trainer indicating that he had to provide also instructions on different 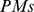 than those selected for this study, e.g. instructions on secondary rowing errors that only have an indirect impact on the rowing performance such as the shoulder posture.
4. The quality of the rowing model and the simulation might not have been high enough to provide the necessary key features that enable a transfer of the improved performance from the simulator to rowing on water. However, the matching of the participants' rankings based on the “mean power” on water and in the simulator suggested a realistic simulation of the interactions (Figure 14). Similarly, results of a previous study on sweep rowing allowed reasonable ranking of athletes according to their experience and preferred oar side (bow/stern) [44]. Furthermore, the “stroke length” and the “overlap of trunk and arms during drive phase” on the simulator were in a similar range as during rowing on water. Moreover, all participants confirmed the realism of the simulator in the questionnaire. Therefore, we believe that the simulator might not only be used to simulate on-water rowing, but also to assess athletes under constant conditions.
5. The choice of the most consistent of three runs for the evaluation of this study might have had an influence on the study results, since the participants could have learnt from one test condition to the next one. However, no learning effect was found in a more detailed analysis. Therefore, the choice of the most consistent runs for evaluation of the study still seems reasonable.
6. Evaluation of biomechanical performance measures was based only on one oar due to a deficiency of one oar sensor on water during retention. In contrary, the independent rowing trainer could analyze the handling and the coordination of both oars. However, significant influences on other aspects than on the oar coordination are not expected.

To summarize the influence of all the previously mentioned points on the expressiveness of the chosen 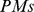, the differences between the ratings based on biomechanical data and the video evaluation were calculated: In most cases (29), one evaluation method revealed a significant increase or decrease in a technical aspect, while the other evaluation method indicated no change. For example, participant “W1” improved her catch angle on water according to the biomechanical performance measures, but the video evaluation did not reveal a performance change. A development in the same direction for both evaluation methods was found in 25 cases for the tests on water. Opposed ratings in the developments on water were only found in two cases. Accordingly, the rating methods did not contradict each other. In contrary, even a clear tendency for a correspondence between the evaluation methods of the 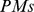 based on data and video evaluation could be found. However, regardless of the way the 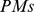 were evaluated, the chosen 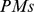 seem to allow only a documentation of basic development in rowing performance. For a detailed insight into performance gains in skilled participants, especially in a setting that is significantly influenced by the environment, the chosen 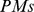 might be limited. Therefore, a thorough evaluation of skilled performance in a complex task in a variable environment still has to rely on conventional rating through a human trainer.

### Transfer Evaluation based on Video Rating of General Aspects

The video rating based on general aspects drew a positive picture of learning and transfer. Regardless of the training group, all participants increased in at least one general aspect, with exception from participant “S4”. This finding confirms that the analysis of the 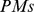 could not explain all important factors in sculling that the trainer could assess. Moreover, the assessment of general aspects revealed that the initial skill level of the participants in the simulator group was on average one point higher than the initial skill level in the participants of the on-water group (Table 4: “overall impression” in the baseline tests). This initial advantage in performance left less space for improvement in the simulator group, especially within the short training period of two weeks. The fact that even participants with advanced skills could improve in the general aspects clearly confirms the effectiveness of simulator training and skill transfer. The basic skill gains measured in the current study are expected to be further increased when additional augmented feedback or modifications in physical parameters are applied, which was shown in previous studies [35], [37].

### Positive Participant Ratings

Realism of the simulator was confirmed in the questionnaire: The average participants' rating of “involvement/control”, “naturalism”, “advantage simulator”, and “interface quality” of the simulator reached 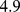 points out of 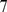 (Figure 15). The participants agreed that the simulator offers a clear advantage over training on water in terms of dependence on good weather conditions and the influence of environmental conditions like wind, waves, and other boats. This reduction of external influences allowed participants of the simulator group to focus more on single technical aspects such as the correct coordination of legs trunk and arms without having to struggle with wind, waves or to worry about a collision with other boats. Moreover, the rowers could train to immerse the oars at the right depth simultaneously on both sides without having to estimate the influence of waves. Therefore, simulator training also indicated its acceptance and relevance for training of real-life tasks and can be seen as a good complement for training in the real environment. However, simulators may not be able to simulate all aspects that are important for a task, e.g. proper handling of rowing equipment or coping with variable environmental conditions must still be learnt under real conditions. In general, all participants were satisfied with the special rowing course (average of 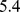 points out of 7 over all questions) and they could all benefit personally (6.5 points out of 7).

### Expected Effects of Larger Group Size

A larger group size would have allowed the use of statistical methods which could reveal between and within group effects and correlations between the biomechanical data and video analysis. However, a larger group size is not expected to change the general results of the current study, i.e. the learning and transfer of skills from the simulator to rowing on water, and the acceptance and relevance of the scull simulator for training of technical aspects for rowing on water.

## Conclusion

In this paper, the skill gain during scull training on a realistic rowing simulator and the transfer of the gained skills to rowing on water were compared to skill gains through rowing training on water. For the current study, only the basic functions of the simulator were used: realistic rendering visual, auditory, and haptic interactions of the rower with the virtual environment. The realism of these interactions with the virtual environment was supported through results from a questionnaire and by similarities in biomechanical performance measures between rowing on water and rowing in the simulator. As transfer to on-water rowing was observed, the presented simulator can now be used as a complementary training tool. Skill gains in the simulator are expected to become more prominent when augmented feedback is added, which was already shown in other transfer studies. Therefore, augmented feedback will be a main focus for future studies on the simulator.

The study also revealed that the applied rendering addressed the key features of rowing. Visual, auditory, and haptic display can now easily be modulated in order to identify their impact on skill gains. Therewith, a cost-effective but still training-efficient training device could be developed filling the gap between high-end simulators as presented here and rowing ergometers.

## Supporting Information

Appendix S1 **Detailed information on the rowing model.**
(PDF)

Figure S1 **Anti windup controller.**
(TIF)

Figure S2 **The CAD-design of the CAVE system in the M3-Lab.** The CAD-design illustrates the scull rowing setup and the coordinate system for the tendon-based parallel robots *R* and the shortened rowing skiff *B*.
(TIF)
