# Supplementary material for: Transfer of Complex Skill Learning from Virtual to Real Rowing
Source: PLoS One. 2013 Dec 20;8(12):e82145. doi: 10.1371/journal.pone.0082145 (PMC3869668; doi:10.1371/journal.pone.0082145)
Supplement: Appendix S1 — Detailed information on the rowing model. (PDF) [file pone.0082145.s001.pdf]

Appendix S1

Detailed Rowing Model

Velocity Model

As a basis for the scull rowing model, the general forms of the equations of variation of linear (1) and angular momentum (2) are used (similar to [1]):

$$m_{Boat}{}^{O}\ddot{\mathbf{x}}_G = {}^{O}\mathbf{f}_G, \tag{1}$$

where m_{Boat} denotes the mass of rower, boat, and oars together, ${}^{O}\ddot{\mathbf{x}}_G$ is the translational acceleration of the boat's center of gravity (COG) G in Euclidean space defined in the global coordinate system $K_O = \{{}^{O}O; {}^{O}x, {}^{O}y, {}^{O}z\}$, and ${}^{O}\mathbf{f}_G$ is the sum of forces applied to the boat.

$${}^{O}\mathbf{T}_B{}^{B}\mathbf{J}_G{}^{B}\mathbf{T}_O{}^{O}\ddot{\omega}_G + {}^{O}\dot{\omega}_G \times {}^{O}\mathbf{T}_B{}^{B}\mathbf{J}_G{}^{B}\mathbf{T}_O{}^{O}\dot{\omega}_G = {}^{O}\tau_G, \tag{2}$$

where ${}^{O}\mathbf{T}_B$ denotes the transformation matrix from the boat-fixed coordinate system $K_B = \{{}^{B}O_G; {}^{B}\mathbf{x}, {}^{B}\mathbf{y}, {}^{B}\mathbf{z}\}$ to global coordinates O, ${}^{B}\mathbf{T}_O$ is the inverse transformation of ${}^{O}\mathbf{T}_B$ from global coordinates O to boat coordinates B, ${}^{B}\mathbf{J}_G$ is the inertia matrix of rower, boat, and oars in G coupled to the boat-fixed coordinate system K_B, ${}^{O}\ddot{\omega}$ and ${}^{O}\dot{\omega}$ are the absolute rotational acceleration and the rotational velocity of the boat in global coordinates O, respectively, and ${}^{O}\tau_G$ is the sum of the torques in global coordinates O applied to the boat with respect to the COG G. To obtain a planar rowing model with the three DOF: ${}^{O}x,\ {}^{O}y,\ and\ {}^{O}\psi$, the following simplifications are applied to equations (1) and (2):

1. the COG G of the system rower-boat-oars is assumed to remain constant
2. the boat-fixed coordinate system K_B is defined in the COG G of the system rower-boat-oars at water level
3. the boat only rotates around the axis ${}^{B}z$ in G, which defines the yaw angle of the boat ${}^{O}\psi$
4. the matrix of inertia of the system rower-boat in K_B is in principal axis form
5. the planar model accounts for the three DOF: ${}^{O}x,\ {}^{O}y,\ and\ {}^{O}\psi$, thus

$${}^{O}\mathbf{x}_G = \begin{bmatrix} {}^{O}x \\ {}^{O}y \\ {}^{O}z \end{bmatrix},\ {}^{O}\omega_G = \begin{bmatrix} {}^{O}\vartheta \\ {}^{O}\varphi \\ {}^{O}\psi \end{bmatrix} \tag{3}$$

are reduced to

$${}^{O}\mathbf{x}_G = \begin{bmatrix} {}^{O}x \\ {}^{O}y \end{bmatrix},\ {}^{O}\omega_G = {}^{O}\psi. \tag{4}$$

Due to the simplifications, equation (1) yields:

$$m_{Boat}\begin{bmatrix} {}^{O}\ddot{x} \\ {}^{O}\ddot{y} \end{bmatrix} = {}^{O}\mathbf{f}_G \tag{5}$$

and equation (2) yields:

$${}^{B}J_{zz}{}^{O}\ddot{\psi} = {}^{O}\tau_{G,z}, \tag{6}$$

where ${}^{B}J_{zz}$ is the moment of inertia of the system rower-boat-oars in yaw direction and ${}^{O}\tau_{G,z}$ is the sum of torques applied to the system rower-boat-oars with respect to G in yaw direction i.e. around axis ${}^{O}z$ in G.

The sum of forces ${}^{O}\mathbf{f}_{G}$ in equation (5) results from the following contributions:

$$ {}^{O}\mathbf{f}_{G} = {}^{O}\mathbf{T}_{B}({}^{B}\mathbf{f}_{prop} - {}^{B}\mathbf{f}_{in} - {}^{B}\mathbf{f}_{boatDrag}). \tag{7} $$

${}^{B}\mathbf{f}_{prop}$ is the sum of propulsive oar forces in boat coordinates B, ${}^{B}\mathbf{f}_{in}$ is the sum of inertial forces resulting from oar and rower movements in boat coordinates B, and ${}^{B}\mathbf{f}_{boatDrag}$ sums the water drag forces acting on the boat hull in boat coordinates B. The sum of propulsive oar forces ${}^{B}\mathbf{f}_{prop}$ can be further divided into propulsive forces acting on the right and the left oar:

$$ {}^{B}\mathbf{f}_{prop} = {}^{B}\mathbf{f}_{prop,r} + {}^{B}\mathbf{f}_{prop,l}. \tag{8} $$

The propulsive forces ${}^{B}\mathbf{f}_{prop,r}$ and ${}^{B}\mathbf{f}_{prop,l}$ directly depend on the interaction between the oars and water and thus are best to be described in their oar fixed coordinate systems $K_{Oar,r}$ and $K_{Oar,l}$, respectively:

$$ \begin{aligned} {}^{B}\mathbf{f}_{prop,r} &= {}^{B}\mathbf{T}_{Oar,r}\,{}^{Oar,r}\mathbf{f}_{prop,r}, \\ {}^{B}\mathbf{f}_{prop,l} &= {}^{B}\mathbf{T}_{Oar,l}\,{}^{Oar,l}\mathbf{f}_{prop,l}. \end{aligned} \tag{9} $$

${}^{B}\mathbf{T}_{Oar,r}$ and ${}^{B}\mathbf{T}_{Oar,l}$ denote the transformations from right- and left oar coordinates into boat coordinates, respectively. Note, that the left oar's coordinate system $K_{Oar,l}$ is chosen to be left-handed. This choice was taken to allow a symmetric definition and use of forces and oar angles on the oars with respect to the symmetry axis ${}^{B}y$ of the boat (Figure 6).These oar angles at the right/left oar are: horizontal oar angle θ_r/θ_r, vertical oar angle δ_r/δ_l, and rotation around the oar's longitudinal axis φ_r/φ_l. In detail, the transformations ${}^{B}\mathbf{T}_{Oar,r}$ and ${}^{B}\mathbf{T}_{Oar,l}$ in equation (9), can be written as:

$$ \begin{aligned} {}^{B}\mathbf{T}_{Oar,r} &= {}^{B}\mathbf{T}_{Oar,r,init}\quad \mathbf{T}_{Oar,r,z}(\theta_r)\quad \mathbf{T}_{Oar,r,x}(\delta_r)\quad \mathbf{T}_{Oar,r,y}(\varphi_r) \\ &= \begin{bmatrix} 0 & 1 & 0 \\ -1 & 0 & 0 \\ 0 & 0 & 1 \end{bmatrix} \begin{bmatrix} c(\theta_r) & -s(\theta_r) & 0 \\ s(\theta_r) & c(\theta_r) & 0 \\ 0 & 0 & 1 \end{bmatrix} \begin{bmatrix} 1 & 0 & 0 \\ 0 & c(\delta_r) & -s(\delta_r) \\ 0 & s(\delta_r) & c(\delta_r) \end{bmatrix} \begin{bmatrix} c(\varphi_r) & 0 & s(\varphi_r) \\ 0 & 1 & 0 \\ -s(\varphi_r) & 0 & c(\varphi_r) \end{bmatrix} \\ {}^{B}\mathbf{T}_{Oar,r} &= \begin{bmatrix} s(\theta_r)c(\varphi_r)+c(\theta_r)s(\delta_r)s(\varphi_r) & c(\theta_r)c(\delta_r) & s(\theta_r)s(\varphi_r)-c(\theta_r)s(\delta_r)c(\varphi_r) \\ -c(\theta_r)c(\varphi_r)+s(\theta_r)s(\delta_r)s(\varphi_r) & s(\theta_r)c(\delta_r) & -c(\theta_r)s(\varphi_r)-s(\theta_r)s(\delta_r)c(\varphi_r) \\ -c(\delta_r)s(\varphi_r) & s(\delta_r) & c(\delta_r)c(\varphi_r) \end{bmatrix} \end{aligned} \tag{10} $$

and

$$ \begin{aligned} {}^{B}\mathbf{T}_{Oar,l} &= {}^{B}\mathbf{T}_{Oar,l,init}\quad \mathbf{T}_{Oar,l,z}(\theta_l)\quad \mathbf{T}_{Oar,l,x}(\delta_l)\quad \mathbf{T}_{Oar,l,y}(\varphi_l) \\ &= \begin{bmatrix} 0 & -1 & 0 \\ -1 & 0 & 0 \\ 0 & 0 & 1 \end{bmatrix} \begin{bmatrix} c(\theta_l) & -s(\theta_l) & 0 \\ s(\theta_l) & c(\theta_l) & 0 \\ 0 & 0 & 1 \end{bmatrix} \begin{bmatrix} 1 & 0 & 0 \\ 0 & c(\delta_l) & -s(\delta_l) \\ 0 & s(\delta_l) & c(\delta_l) \end{bmatrix} \begin{bmatrix} c(\varphi_l) & 0 & s(\varphi_l) \\ 0 & 1 & 0 \\ -s(\varphi_l) & 0 & c(\varphi_l) \end{bmatrix} \\ {}^{B}\mathbf{T}_{Oar,l} &= \begin{bmatrix} -s(\theta_l)c(\varphi_l)-c(\theta_l)s(\delta_l)s(\varphi_l) & -c(\theta_l)c(\delta_l) & -s(\theta_l)s(\varphi_l)+c(\theta_l)s(\delta_l)c(\varphi_l) \\ -c(\theta_l)c(\varphi_l)+s(\theta_l)s(\delta_l)s(\varphi_l) & s(\theta_l)c(\delta_l) & -c(\theta_l)s(\varphi_l)-s(\theta_l)s(\delta_l)c(\varphi_l) \\ -c(\delta_l)s(\varphi_l) & s(\delta_l) & c(\delta_l)c(\varphi_l) \end{bmatrix} \end{aligned} \tag{11} $$

The components of the oar blade forces in oar coordinates from equation (9) can be defined as:

$$ {}^{Oar,r}\mathbf{f}_{prop,r} = \begin{bmatrix} {}^{Oar,r}\mathbf{f}_{\theta_r} \\ {}^{Oar,r}\mathbf{f}_{l_r} \\ {}^{Oar,r}\mathbf{f}_{\delta_r} \end{bmatrix},\ {}^{Oar,l}\mathbf{f}_{prop,l} = \begin{bmatrix} {}^{Oar,l}\mathbf{f}_{\theta_l} \\ {}^{Oar,l}\mathbf{f}_{l_l} \\ {}^{Oar,l}\mathbf{f}_{\delta_l} \end{bmatrix}. \tag{12} $$

In order to calculate the 3DOF planar boat movement, only the horizontal normal force components ${}^{Oar,r}\mathbf{f}_{\theta_r}$ and ${}^{Oar,l}\mathbf{f}_{\theta_l}$ of the propulsion forces at the oars are taken into account. Thus, equation (12) can be rewritten as:

$$ {}^{Oar,r}\mathbf{f}_{prop,r} = \begin{bmatrix} {}^{Oar,r}\mathbf{f}_{\theta_r}\ s(\theta_r) \\ -{}^{Oar,r}\mathbf{f}_{\theta_r}\ c(\theta_r) \end{bmatrix},\ {}^{Oar,l}\mathbf{f}_{prop,l} = \begin{bmatrix} -{}^{Oar,l}\mathbf{f}_{\theta_l}\ s(\theta_l) \\ -{}^{Oar,l}\mathbf{f}_{\theta_l}\ c(\theta_l) \end{bmatrix}. \tag{13} $$

This simplification has hardly any effect on the planar behaviour of the boat, since the other two force directions (${}^{Oar}y$, ${}^{Oar}z$) indicate significantly lower forces, compared to ${}^{Oar,r}\mathbf{f}_{\theta_r}$ and ${}^{Oar,l}\mathbf{f}_{\theta_l}$. However, for haptic rendering of the oar-water interaction, also oar forces in direction ${}^{Oar}z$ are displayed. Forces in direction of ${}^{Oar}y$ are not

rendered since the fixation of the oars in the simulator's oarlocks do not allow movements in this direction. All force components acting on their respective oar will be derived in detail in the subsection "Force Model".

The second type of forces in equation (7) contributing to the boat movement are the inertia forces ${}^B\mathbf{f}_{in}$. ${}^B\mathbf{f}_{in}$ can be further divided into inertial forces due to movement of the right- ${}^B\mathbf{f}_{in,Oar,r}$ and left oar ${}^B\mathbf{f}_{in,Oar,l}$ and inertial forces due to the rower's movement ${}^B\mathbf{f}_{in,rower}$:

$$ {}^B\mathbf{f}_{in} = {}^B\mathbf{f}_{in,Oar,r} + {}^B\mathbf{f}_{in,Oar,l} + {}^B\mathbf{f}_{in,rower}. \tag{14} $$

Where the oar inertia forces can be obtained through multiplication of the oar mass m_{Oar} with the acceleration of the oar's COG $\mathbf{l}_{OL2COG}$:

$$ \begin{gathered} {}^B\mathbf{f}_{in,Oar,r} = m_{Oar}\frac{d^2({}^B\mathbf{T}_{Oar,r}\,\mathbf{l}_{OL2COG})}{dt^2}, \\ {}^B\mathbf{f}_{in,Oar,l} = m_{Oar}\frac{d^2({}^B\mathbf{T}_{Oar,l}\,\mathbf{l}_{OL2COG})}{dt^2} \\ \text{with} \\ \mathbf{l}_{OL2COG} = \begin{bmatrix} 0\ \text{m} \\ 0.565\ \text{m} \\ 0\ \text{m} \end{bmatrix}, \\ m_{Oar} = 1.2\ \text{kg}. \end{gathered} \tag{15} $$

The rower's inertial forces are obtained by multiplication of the rower's mass m_{Rower} times the measured seat acceleration ${}^B\ddot{y}_{Seat}$. The accelerations in other directions are not considered since the seat only moves in ${}^By$ direction:

$$ {}^B\mathbf{f}_{in,rower} = m_{Rower}\begin{bmatrix} 0 \\ {}^B\ddot{y}_{Seat} \\ 0 \end{bmatrix}. \tag{16} $$

The third type of forces in equation (7) influencing the boat movement are the drag forces ${}^B\mathbf{f}_{boatDrag}$ that always act opposed to the boat movement:

$$ \begin{gathered} {}^B\mathbf{f}_{boatDrag} = \begin{bmatrix} C_{B,x}\ sign({}^B\dot{x}_G)\ {}^B\dot{x}_G{}^2 \\ C_{B,y}\ sign({}^B\dot{y}_G)\ {}^B\dot{y}_G{}^2 \\ 0 \end{bmatrix} \\ \text{with} \\ C_{B,x} = 316\ \frac{\text{kg}}{\text{m}}, \\ C_{B,y} = 3.16\ \frac{\text{kg}}{\text{m}}. \end{gathered} \tag{17} $$

$C_{B,x}$ and $C_{B,y}$ denote the boat drag coefficients in directions ${}^B\mathbf{x}$ and ${}^B\mathbf{y}$, respectively. The boat drag coefficient $C_{B,x}$ was determined heuristically, whereas $C_{B,y}$ was taken from literature [2].

The sum of torques applied to the boat ${}^O\tau_G$ according to the equation of variation of angular momentum (2) can be divided similarly to the sum of forces in equation (7):

$$ {}^O\tau_{G,z} = ({}^O\mathbf{T}_B({}^B\tau_{prop} - {}^B\tau_{in} - {}^B\tau_{boatDrag}))_z. \tag{18} $$

${}^B\tau_{prop}$ is the sum of torques in boat coordinates B applied to the boat through propulsive oar forces, ${}^B\tau_{in}$ is the sum of inertial torques resulting from oar movements in boat coordinates B, and ${}^B\tau_{boatDrag}$ sums the water drag

torques acting on the boat hull in boat coordinates B. The sum of torques due to propulsive oar forces ${}^{B}\tau_{prop}$ can be derived from the oar forces that induce torques around the boat's COG G:

$$\begin{gathered} {}^{B}\tau_{prop} = {}^{B}\tau_{prop,r} + {}^{B}\tau_{prop,l} \\ \text{with} \\ {}^{B}\tau_{prop,r} = {}^{B}\mathbf{x}_{EE,r} \times {}^{B}\mathbf{f}_{prop,r}, \\ {}^{B}\tau_{prop,l} = {}^{B}\mathbf{x}_{EE,l} \times {}^{B}\mathbf{f}_{prop,l}. \end{gathered} \tag{19}$$

${}^{B}\mathbf{x}_{EE,r}$ and ${}^{B}\mathbf{x}_{EE,l}$ are the vectors that describe the oar position i.e. the end-effector position of the right and the left oar, respectively. Both vectors ${}^{B}\mathbf{x}_{EE,r}$ and ${}^{B}\mathbf{x}_{EE,l}$ are expressed in boat coordinates B with respect to the point G around which the boat turns:

$$\begin{gathered} {}^{B}\mathbf{x}_{EE,r} = {}^{B}\mathbf{T}_{Oar,r}\, \mathbf{l}_{OL2EE} + {}^{B}\mathbf{x}_{OL,r} \\ {}^{B}\mathbf{x}_{EE,l} = {}^{B}\mathbf{T}_{Oar,r}\, \mathbf{l}_{OL2EE} + {}^{B}\mathbf{x}_{OL,l}, \\ \text{with} \\ \mathbf{l}_{OL2EE} = \begin{bmatrix} 0\text{ m} \\ 1.155\text{ m} \\ 0\text{ m} \end{bmatrix}, \\ {}^{B}\mathbf{x}_{OL,r} = \begin{bmatrix} 0.8\text{ m} \\ 0.75\text{ m} \\ 0.25\text{ m} \end{bmatrix}, \\ {}^{B}\mathbf{x}_{OL,l} = \begin{bmatrix} -0.8\text{ m} \\ 0.75\text{ m} \\ 0.26\text{ m} \end{bmatrix}. \end{gathered} \tag{20}$$

$\mathbf{l}_{OL2EE}$ denotes the outboard oar length i.e. the vector from oar lock to the end-effector EE in oar coordinates $K_{Oar,r}$ and $K_{Oar,l}$ for the right and the left oar, respectively. ${}^{B}\mathbf{x}_{OL,r}$ and ${}^{B}\mathbf{x}_{OL,l}$ are the positions of the right and left oar lock, respecitvely, expressed in the boat coordinate system K_B. The positions of the oar locks correspond to the centers of the oar coordinate systems.

The second type of torques contributing to the boat movement according to equation (7), are the inertia torques ${}^{B}\tau_{in}$:

$$\begin{gathered} {}^{B}\tau_{in} = {}^{B}\tau_{in,r} + {}^{B}\tau_{in,l} \\ \text{with} \\ {}^{B}\tau_{in,r} = {}^{B}\mathbf{x}_{EE,r} \times {}^{B}\mathbf{f}_{in,Oar,r}, \\ {}^{B}\tau_{in,l} = {}^{B}\mathbf{x}_{EE,l} \times {}^{B}\mathbf{f}_{in,Oar,l}. \end{gathered} \tag{21}$$

The third torque type in equation (7) that influences the movement of the boat is the torque due do drag forces ${}^{B}\tau_{boatDrag}$ acting on the boat. This drag torque results from two effects:

$${}^{B}\tau_{boatDrag} = {}^{B}\tau_{boatDrag,vel} + {}^{B}\tau_{boatDrag,fin}. \tag{22}$$

The first effect is an angular velocity-dependent drag torque ${}^{B}\tau_{boatDrag,vel}$ that damps boat rotations. The second effect is a stabilizing torque induced by the fin at the bottom of the boat ${}^{B}\tau_{boatDrag,fin}$ that counteracts yawing of

the boat.

$$
{}^{B}\tau_{boatDrag,vel} = \begin{bmatrix} 0 \\ 0 \\ \tau_{B,\psi,\dot{\psi}} \end{bmatrix},
$$
$$
{}^{B}\tau_{boatDrag,fin} = \begin{bmatrix} 0 \\ 0 \\ \tau_{B,\psi,fin} \end{bmatrix}
$$

with

$$
\begin{aligned}
\tau_{B,\psi,\dot{\psi}} &= C_{B,\psi,\dot{\psi}} sign({}^{B}\dot{\psi}){}^{B}\dot{\psi}^{2}, \\
\tau_{B,\psi,fin} &= C_{B,\psi,fin} sign({}^{B}\dot{\psi}) \\
&((r_{G2Fin}{}^{B}\dot{\psi})^{2} + {}^{B}\dot{y}^{2}) \\
&atan(\frac{(r_{G2Fin}{}^{B}\dot{\psi})^{2}}{{}^{B}\dot{y}^{2}}), \\
C_{B,\psi,\dot{\psi}} &= 100 \text{ Nms}^{2}, \\
C_{B,\psi,fin} &= 5000 \text{ m}, \\
r_{G2Fin} &= 3 \text{ m}.
\end{aligned} \tag{23}
$$

Here, $C_{B,\psi,\dot{\psi}}$ denotes the rotational boat drag coefficient in ψ direction, $C_{B,\psi,fin}$ is the fin drag coefficient in yaw direction ψ, and r_{G2Fin} is the distance of the fin on the keel from the origin of the boat coordinate system G in stern direction ${}^{B}\mathbf{y}$. The stabilizing torque induced by the fin ${}^{B}\tau_{boatDrag,fin}$ increases with increased boat velocity in direction ${}^{B}\mathbf{y}$ and increased yaw velocity $\dot{\psi}$. The two coefficients $C_{B,\psi,fin}$ and r_{G2Fin} enable a simple adaption of the amount of stabilizing torque. Equation (23) and the corresponding coefficients were established heuristically.

In order to haptically simulate the oar forces, the boat's velocity relative to the water has to be calculated: ${}^{B}\dot{\psi}(t)$, ${}^{B}\dot{x}(t)$, and ${}^{B}\dot{y}(t)$. For the graphical scenario, the planar motion of the boat has to be calculated in global coordinates O: ${}^{O}\psi(t)$, ${}^{O}\dot{\psi}(t)$, ${}^{O}x(t)$, ${}^{O}\dot{x}(t)$, ${}^{O}y(t)$, and ${}^{O}\dot{y}(t)$. To calculate the angular movement $\psi(t)$ and its derivation $\dot{\psi}(t)$, the simplified equation of variation (6) is resolved with respect to ${}^{O}\ddot{\psi}(t)$ and integrated over time:

$$
\begin{aligned}
{}^{O}\dot{\psi}(t) &= \int_{0}^{t} \frac{{}^{O}\tau_{G,z}}{{}^{B}J_{zz}} dt + {}^{O}\dot{\psi}(t=0), \\
{}^{O}\psi(t) &= \int_{0}^{t} (\int_{0}^{t} \frac{{}^{O}\tau_{G,z}}{{}^{B}J_{zz}} dt + {}^{O}\dot{\psi}(t=0)) dt + {}^{O}\psi(t=0), \\
{}^{O}\dot{\psi}(t) &= {}^{B}\dot{\psi}(t), {}^{O}\psi(t) = {}^{B}\psi(t).
\end{aligned} \tag{24}
$$

Knowing the yaw angle ψ, the rotation matrix ${}^{O}\mathbf{T}_{B}$ and its inverse ${}^{B}\mathbf{T}_{O}$ can be calculated:

$$
{}^{O}\mathbf{T}_{B} = \begin{bmatrix} c(\psi) & -s(\psi) & 0 \\ s(\psi) & c(\psi) & 0 \\ 0 & 0 & 1 \end{bmatrix}. \tag{25}
$$

Solving the simplified equation of variation of linear momentum (5) for ${}^{O}\ddot{x}(t)$ and ${}^{O}\ddot{y}(t)$ and integrating over time yields:

$$
\begin{aligned}
\begin{bmatrix} {}^{O}\dot{x}(t) \\ {}^{O}\dot{y}(t) \end{bmatrix} &= \int_{0}^{t} \frac{{}^{O}\mathbf{f}}{m_{Boat}} dt + \begin{bmatrix} {}^{O}\dot{x}(t=0) \\ {}^{O}\dot{y}(t=0) \end{bmatrix}, \\
\begin{bmatrix} {}^{O}x(t) \\ {}^{O}y(t) \end{bmatrix} &= \int_{0}^{t} (\int_{0}^{t} \frac{{}^{O}\mathbf{f}}{m_{Boat}} dt + \begin{bmatrix} {}^{O}\dot{x}(t=0) \\ {}^{O}\dot{y}(t=0) \end{bmatrix}) dt + \begin{bmatrix} {}^{O}x(t=0) \\ {}^{O}y(t=0) \end{bmatrix}.
\end{aligned} \tag{26}
$$

To obtain the relative boat movement ${}^{B}x(t)$ and ${}^{B}y(t)$, the simplified equation of variation of linear momentum (5) has first to be transformed to boat coordinates B before it can be treated similar to equations (26):

$$\begin{bmatrix} {}^{B}\dot{x}(t) \\ {}^{B}\dot{y}(t) \end{bmatrix} = \int_0^t \frac{{}^{B}\mathbf{T}_O {}^{O}\mathbf{f}}{m_{Boat}} dt + \begin{bmatrix} {}^{B}\dot{x}(t=0) \\ {}^{B}\dot{y}(t=0) \end{bmatrix}. \tag{27}$$

Without loss of generality, all initial velocities and positions in equations (24,26,27) were set to zero:

$${}^{O}\dot{\psi}(t=0) = 0, {}^{O}\psi(t=0) = 0, \tag{28}$$

$${}^{O}\dot{x}(t=0) = 0, {}^{O}\dot{y}(t=0) = 0, \tag{29}$$

$${}^{O}x(t=0) = 0, {}^{O}y(t=0) = 0, \tag{30}$$

$${}^{B}\dot{x}(t=0) = 0, {}^{B}\dot{y}(t=0) = 0. \tag{31}$$

However, for the study described in this paper, the boat is restricted to remain on a lane between two lines of buoyes. The lane is $6\ \mathrm{km}$ long and $14\ \mathrm{m}$ wide (Figure 4). The restriction to the lane has several reasons: the rowers should feel like being in an environment where professional rowing races are carried out and they should not be able to row outside of the area that is defined as water in the graphics scenario. For safety reasons also the velocities are saturated. Therefore, the calculations of the boat movement in equations (24,26,27) have to be modified. The restrictions are implemented in form of a saturation embedded inside an anti-windup loop (Figure S1).In the following, the function of the anti-windup controller will be abbreviated in the following form:

$$u_{out} = Antiwindup(P, u_{min}, u_{max})\dot{u}_{in}. \tag{32}$$

Where P denotes the proportional gain of the feed-back part in the controller, and u_{min} and u_{max} are the lower and upper limit of the saturation, respectively. In a modified form, equation (24) of angular movement $\psi(t)$ and its derivation $\dot{\psi}(t)$ now yields:

$$\begin{gathered} {}^{O}\dot{\psi}_{sat}(t) = Antiwindup(0.3\ \tfrac{1}{\mathrm{s}}, -10\ \tfrac{\circ}{\mathrm{s}}, 10\ \tfrac{\circ}{\mathrm{s}}) \frac{{}^{O}\tau_{G,z}}{{}^{B}J_{zz}}, \\ {}^{O}\psi_{sat}(t) = Antiwindup(0.3\ \tfrac{1}{\mathrm{s}}, -50\ \tfrac{\circ}{\mathrm{s}}, 50\ \tfrac{\circ}{\mathrm{s}}) {}^{O}\dot{\psi}_{sat}(t), \\ {}^{O}\dot{\psi}_{sat}(t) = {}^{B}\dot{\psi}_{sat}(t), {}^{O}\psi_{sat}(t) = {}^{B}\psi_{sat}(t). \end{gathered} \tag{33}$$

Thus, the transformation from boat- to global coordinates from equation (25) has also to be modified:

$${}^{O}\mathbf{T}_B(\psi_{sat}) = \begin{bmatrix} c(\psi_{sat}) & -s(\psi_{sat}) & 0 \\ s(\psi_{sat}) & c(\psi_{sat}) & 0 \\ 0 & 0 & 1 \end{bmatrix}. \tag{34}$$

The linear motion in global coordinates O is adapted from equation (26) and now yields:

$$\begin{gathered} {}^{O}\dot{x}_{sat}(t) = Antiwindup(0.3\ \tfrac{1}{\mathrm{s}}, -2\ \tfrac{\mathrm{m}}{\mathrm{s}}, 2\ \tfrac{\mathrm{m}}{\mathrm{s}}) \frac{{}^{O}\mathbf{f}_x}{m_{Boat}}, \\ {}^{O}\dot{y}_{sat}(t) = Antiwindup(0.3\ \tfrac{1}{\mathrm{s}}, -8\ \tfrac{\mathrm{m}}{\mathrm{s}}, 2\ \tfrac{\mathrm{m}}{\mathrm{s}}) \frac{{}^{O}\mathbf{f}_y}{m_{Boat}}, \\ {}^{O}x_{sat}(t) = Antiwindup(0.3\ \tfrac{1}{\mathrm{s}}, -7\ \mathrm{m}, 7\ \mathrm{m}) {}^{O}\dot{x}_{sat}(t), \\ {}^{O}y_{sat}(t) = Antiwindup(0.3\ \tfrac{1}{\mathrm{s}}, -5000\ \mathrm{m}, 1000\ \mathrm{m}) {}^{O}\dot{y}_{sat}(t). \end{gathered} \tag{35}$$

Since the movement of the boat is restricted through saturations, also all other movements that are needed in further steps of the rowing model have to incorporate these saturations. This is the case for the relative velocity between water and the right ${}^{O}\dot{\mathbf{x}}_{EE,r,sat}$ and left ${}^{O}\dot{\mathbf{x}}_{EE,r,sat}$ oar. These two velocities are needed to render the oar forces at the corresponding oar:

$$\begin{aligned} {}^{O}\dot{\mathbf{x}}_{EE,r,sat} &= \frac{d^{O}\mathbf{x}_{sat}(t)}{dt} + \frac{d^{B}\mathbf{x}_{EE,r}}{dt}, \\ {}^{O}\dot{\mathbf{x}}_{EE,l,sat} &= \frac{d^{O}\mathbf{x}_{sat}(t)}{dt} + \frac{d^{B}\mathbf{x}_{EE,l}}{dt}. \end{aligned} \tag{36}$$

However, the relative oar velovity calculated in this way would lead to a change of oar forces as soon as the boat's pose, i.e. position and rotation, is saturated. Thus, for safety reasons, and also to not disturb the rowers during training, the boat velocity is not derived through differentiation from the saturated position, but directly taken from the results in equation (35):

$$\begin{aligned} {}^{O}\dot{\mathbf{x}}_{EE,r,sat} &= {}^{O}\dot{\mathbf{x}}_{sat}(t) + \frac{d^{B}\mathbf{x}_{EE,r}}{dt}, \\ {}^{O}\dot{\mathbf{x}}_{EE,l,sat} &= {}^{O}\dot{\mathbf{x}}_{sat}(t) + \frac{d^{B}\mathbf{x}_{EE,l}}{dt}. \end{aligned} \tag{37}$$

In this way, a rower does not feel when he/she touches the border of the lane with the boat and is not disturbed in the rowing performance. However, the rower would remark that the rowing scenario does not move anymore in the saturated direction and thus the visual flow abruptely changes. This visual cue can help the rower to correct the boat movement and get back onto the lane.

Force Model

The force model describes how the oar forces ${}^{Oar,r}\mathbf{f}_{prop,r}$ and ${}^{Oar,l}\mathbf{f}_{prop,l}$ in equation (12) are obtained. These oar forces are rendered by the robot to provide haptic interaction for the user and also to propel the virtual boat through the virtual scenario.

The two oar forces ${}^{Oar}\mathbf{f}_{\theta}$ and ${}^{Oar}\mathbf{f}_{\delta}$ can be adapted from equations (1) and (4) from [3] in the following way using the 3 DOF velocity model:

$$\begin{aligned} {}^{Oar}\mathbf{f}_{\theta} &= -2C_{O_L}^{max} \left| {}^{O}\dot{\mathbf{x}}_{EE,sat} \right|^2 \sin\alpha, \\ {}^{Oar}\mathbf{f}_{\delta} &= -2C_{O_L}^{max} (|\mathbf{l}_{OL2EE}|\, \dot{\delta})^2, \\ &\text{with} \\ \sin\alpha &= \frac{-{}^{O}\mathbf{T}_{Oar}\, {}^{Oar}\mathbf{x}\, {}^{O}\dot{\mathbf{x}}_{EE,sat}}{|{}^{O}\dot{\mathbf{x}}_{EE,sat}|}, \\ C_{O_L}^{max} &= 29.35 \frac{\text{kg}}{\text{m}}. \end{aligned} \tag{38}$$

Hereby, α denotes the angle of attack and $C_{O_L}^{max}$ is a resistance coefficient for the oar in water. Note that equation (38) takes an inversion of the force sign, since the forces applied from water to the oars has to be haptically rendered by the robots and not the reaction force of the oars on water. The third force component of ${}^{Oar,r}\mathbf{f}_{prop,r}$ is the force ${}^{Oar}\mathbf{f}_{l}$ in direction of the oar ${}^{Oar}y$. This force component does not have to be rendered because the fixation of the oar in the oar lock does not transfer this force to the rower.

However, there is one more force acting on the oar that has not been introduced so far: the buoyancy force ${}^{O}\mathbf{f}_{buyoncy}$ of the oar. This force makes the oars float on the water surface. Thus, the rowers can haptically determine at which heights the oars are immersed or out of water. ${}^{O}\mathbf{f}_{buyoncy}$ is implemented for haptic rendering

in the following form and always points in ${}^{O}z$ direction:

$$
{}^{O}\mathbf{f}_{buyoncy} = \begin{cases} \rho g \; V_{blade} \frac{\delta_{touch}-\delta}{||\delta_{touch}|-|\delta_{in}||} & \delta \leq \delta_{touch} \\ 0 & \text{else} \end{cases}
$$

$$
\begin{aligned}
&\text{with} \\
\delta_{touch} &= -5.16^{\circ}, \\
\delta_{in} &= -11^{\circ}, \\
\rho &= 998.2\frac{\mathrm{kg}}{\mathrm{m}^3}, \\
g &= 9.81\frac{\mathrm{m}}{\mathrm{s}^2}, \\
V_{blade} &\approx 0.0015\mathrm{m}^3.
\end{aligned} \tag{39}
$$

ρ denotes the relative density of water at $15^{\circ}\mathrm{C}$, δ_{touch} is the vertical oar angle δ, where the flat oar ($\varphi = 0^{\circ}$) touches the water surface, δ_{in} is the vertical oar angle δ, where the flat oar ($\varphi = 0^{\circ}$) is completely immersed in water, g is the earth's gravitational acceleration, and V_{blade} is the approximate volume of the entirely immersed oar blade. The vertical oar angles δ_{touch} and δ_{in}, were determined heuristically together with professional rowers.

For haptic rendering, the oar force ${}^{Oar}\mathbf{f}_{prop}$ has to be transformed into the common coordinate system of both robots $K_R = \{{}^{R}O; {}^{R}\mathbf{x}, {}^{R}\mathbf{y}, {}^{R}\mathbf{z}\}$ (Figure S2).${}^{O}\mathbf{f}_{buyoncy}$ is already given in global coordinates. The global coordinate system K_O has the same orientation as the robot coordinate system K_R. Thus, in our case, the transformation of ${}^{Oar}\mathbf{f}_{prop}$ from oar coordinates to robot coordinates can also be performed using the rotation matrix from oar coordinates to robot coordinates:

$$
\begin{aligned}
{}^{R}\mathbf{f}_{prop,r} &= {}^{O}\mathbf{T}_{Oar,r}\,{}^{Oar,r}\mathbf{f}_{prop,r} \\
&= {}^{R}\mathbf{T}_{Oar,r}\,{}^{Oar,r}\mathbf{f}_{prop,r}, \\
{}^{R}\mathbf{f}_{prop,l} &= {}^{O}\mathbf{T}_{Oar,l}\,{}^{Oar,l}\mathbf{f}_{prop,l} \\
&= {}^{R}\mathbf{T}_{Oar,l}\,{}^{Oar,l}\mathbf{f}_{prop,l}.
\end{aligned} \tag{40}
$$

The forces that have to be rendered at the right and left end-effector by the right and the left robot, respectively, are obtained in the following way:

$$
\begin{aligned}
{}^{R}\mathbf{f}_{dem,r} &= {}^{R}\mathbf{T}_{Oar,r}\,{}^{Oar,r}\mathbf{f}_{prop,r} + \begin{bmatrix} 0 \\ 0 \\ {}^{O}\mathbf{f}_{buyoncy,r} \end{bmatrix}, \\
{}^{R}\mathbf{f}_{dem,l} &= {}^{R}\mathbf{T}_{Oar,l}\,{}^{Oar,l}\mathbf{f}_{prop,l} + \begin{bmatrix} 0 \\ 0 \\ {}^{O}\mathbf{f}_{buyoncy,l} \end{bmatrix}.
\end{aligned} \tag{41}
$$

where ${}^{R}\mathbf{f}_{dem,r}$ denotes the rendering force of the right robot in the robot coordinate system K_R, and ${}^{R}\mathbf{f}_{dem,l}$ is the rendering force of the left robot in the robot coordinate system K_R.

References

1. Azcueta R (2002) Computation of turbulent free-surface flows around ships and floating bodies. Ship Technology Research 49: 46–69.

2. Cabrera D, Ruina A, Kleshnev V (2006) A simple 1+ -dimensional model of rowing mimics observed forces and motions. Human Movement Science 25: 192–220.

3. Rauter G, von Zitzewitz J, Duschau-Wicke A, Vallery H, Riener R (2010) A tendon based parallel robot applied to motor learning in sports. In: Proceedings of the IEEE BioRob. Tokyo, Japan.
