# Supplementary figures and images for: Transfer of Complex Skill Learning from Virtual to Real Rowing
Source: PLoS One. 2013 Dec 20;8(12):e82145. doi: 10.1371/journal.pone.0082145 (PMC3869668; doi:10.1371/journal.pone.0082145)

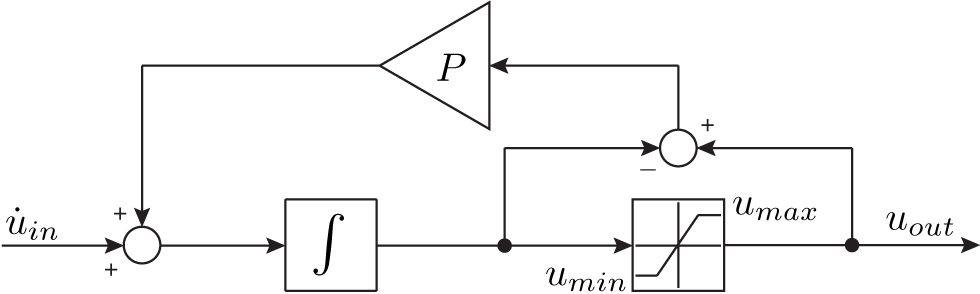

Supplement: Figure S1 — Anti windup controller. (TIF) [file pone.0082145.s002.tif]

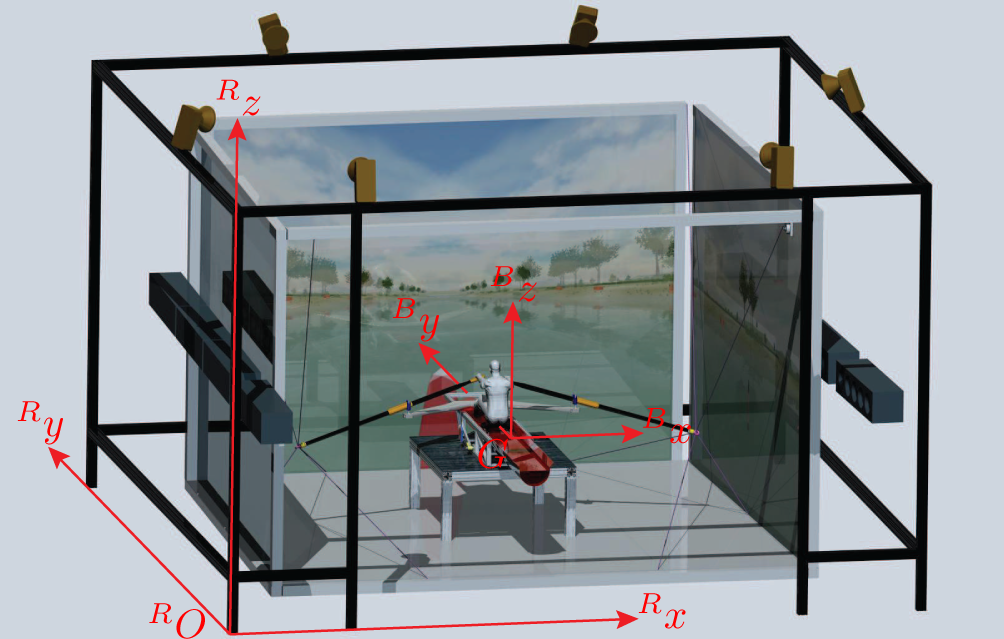

Supplement: Figure S2 — The CAD-design of the CAVE system in the M3-Lab. The CAD-design illustrates the scull rowing setup and the coordinate system for the tendon-based parallel robots R and the shortened rowing skiff B. (TIF) [file pone.0082145.s003.tif]
